# Profiling and targeting cancer stem cell signaling pathways for cancer therapeutics

**DOI:** 10.3389/fcell.2023.1125174

**Published:** 2023-05-25

**Authors:** Mia C. Borlongan, Hongbin Wang

**Affiliations:** ^1^ Master Program of Pharmaceutical Science College of Graduate Studies, Elk Grove, CA, United States; ^2^ Department of Pharmaceutical and Biomedical Sciences College of Pharmacy, Elk Grove, CA, United States; ^3^ Department of Basic Science College of Medicine, California Northstate University, Elk Grove, CA, United States

**Keywords:** cancer stem cells (CSCs), Wnt, TGFβ, Notch, JAK-STAT, hedgehog, VEGF, therapeutics

## Abstract

Tumorigenic cancer stem cells (CSCs) represent a subpopulation of cells within the tumor that express genetic and phenotypic profiles and signaling pathways distinct from the other tumor cells. CSCs have eluded many conventional anti-oncogenic treatments, resulting in metastases and relapses of cancers. Effectively targeting CSCs’ unique self-renewal and differentiation properties would be a breakthrough in cancer therapy. A better characterization of the CSCs’ unique signaling mechanisms will improve our understanding of the pathology and treatment of cancer. In this paper, we will discuss CSC origin, followed by an in-depth review of CSC-associated signaling pathways. Particular emphasis is given on CSC signaling pathways’ ligand-receptor engagement, upstream and downstream mechanisms, and associated genes, and molecules. Signaling pathways associated with regulation of CSC development stand as potential targets of CSC therapy, which include Wnt, TGFβ (transforming growth factor-β)/SMAD, Notch, JAK-STAT (Janus kinase-signal transducers and activators of transcription), Hedgehog (Hh), and vascular endothelial growth factor (VEGF). Lastly, we will also discuss milestone discoveries in CSC-based therapies, including pre-clinical and clinical studies featuring novel CSC signaling pathway cancer therapeutics. This review aims at generating innovative views on CSCs toward a better understanding of cancer pathology and treatment.

## 1 Introduction

Although cancer death rates decreased by 27% from 200.8 deaths in 1999 to 146.2 deaths in 2019 per 100,000 population, cancer remains the second leading cause of death, after heart disease, in the United States (Siegel et al., 2021). In 2019, more male cancer patients (315,876) succumbed to death than females (283,725), with increased mortality rates in males (172.9 deaths per 100,000 population) than females (126.2 deaths per 100,000 population) (Siegel et al., 2021). The economic burden to the society of cancer care for patients stands at $21 billion in the United States in 2019 (Ma and Richardson, 2022). Approximately 16.9 million Americans displayed a history of cancer in 2019 and by 2050 it is predicted the total number of incident cases will increase by almost 50% (Weir et al., 2021). In the world, prostate, colorectum, and melanoma of the skin correspond to the top three prevalent cancers in males, while breast, uterine corpus, and colorectum represent the most prevalent cancers in females (Cao et al., 2021). The deadliest cancer types across all races include lung and bronchus, colorectal, pancreatic, and breast cancers, which account for nearly 50% of all cancer deaths (Cao et al., 2021). About one-third of cancer deaths implicate risk factors, such as tobacco use, high body mass index, alcohol use, low fruit and vegetable intake, and lack of physical activity. Unfortunately, these risk factors skew towards increased morbidity among low and middle income populations due to late-stage presentation and lack of access to diagnosis and treatment (Dieteren and Bonfrer, 2021). Despite significant advances in our scientific understanding of the disease and improved clinical care, cancer treatments remain a significant unmet need. Here, we explore the concept of cancer stem cells (CSCs), which exist as a small subpopulation of cells within tumors exhibiting self-renewal, differentiation, and tumorigenicity (Pattabiraman and Weinberg, 2014; Müller et al., 2020; Chen et al., 2021a). Noteworthy, CSCs elude conventional chemotherapy and radiation treatment, suggesting that CSCs may very well be the origin of cancer metastasis (Chen et al., 2021a). The structure of CSCs also varies based on type of cancer, genetics, and epigenetics, and can differentiate into multiple cell types (e.g., fibroblasts, pericytes, endothelial cells, etc.) (Pattabiraman and Weinberg, 2014; Chen et al., 2021a). Recognizing these unique properties of CSCs opens novel avenues for a better understanding of cancer pathology and its treatment. Indeed, recent innovative cancer treatments utilize a CSC-based treatment platform. To this end, we discuss new lines of investigations probing CSC origin and microenvironment, detailing CSC genotypic and phenotypic profiles and signaling pathways that directly target tumor-forming CSCs.

## 2 Defining cancer stem cells

Cancer is defined as a malignant growth or tumor resulting from the division of abnormal cells (Cowan et al., 2022). The cells within the tumor microenvironment (TME) display varying levels of proliferation and differentiation leading to functional heterogeneity of tumor cells (Pizer et al., 1998; Naruse et al., 2021). Such functional heterogeneity among tumor cells begs the question on the origin of these cells. The conventional concept of abnormal cell division stipulates that cancer is caused by changes to DNA or genetic mutations (Germain et al., 2022). A cell becomes cancerous via multiple alterations to the cell’s DNA sequence (Lasorsa et al., 2022; Valle-Mendiola et al., 2022), with the mutations capable of arising from any cell in the body, thereafter, leading to cancer (Noble, 2021). The stochastic model or clonal evolution model of cancer origin states that all cells exhibit the capacity of self-renewal or differentiation, which becomes uncontrolled through genetic mutations, propelling the growth of heterogenic tumor cells (Odoux et al., 2008; Greaves and Maley, 2012). A newly proposed model of cancer origin advances the notion that a small population of stem cells exists within the tumor that express tumorigenic characteristics, namely the cells’ enduring ability to self-renew and to differentiate into cancer cells, thereby specifically modulating cancer onset and progression. These cancer-driving cells are the CSCs, and fittingly enough this concept of cancer origin is called the CSC model (Kreso and Dick, 2014). Recognizing that CSCs and non-CSCs represent distinct compartments within the tumor provides the functional heterogeneity of the tumor.

In parallel to the aforementioned models of cancer origin (i.e., conventional model versus the stochastic model or clonal evolution model), there are multiple noteworthy theories about origins for CSCs that have garnered compelling evidence. CSCs were first discovered in 1994 in acute myeloid leukemia (AML) rodents displaying severe combined immunodeficient (SCID) (Pattabiraman and Weinberg, 2014; Chen et al., 2021a). These unique cells exhibit not only self-renewing and tumor initiating capabilities akin to stem cells, but they also possess features of asymmetric division (Pattabiraman and Weinberg, 2014). The theory of asymmetric division states that one lineage of CSCs has self-renewing capabilities while the other lineage will lose their stemness properties and become epithelial cells which form the bulk of the tumor (Pattabiraman and Weinberg, 2014). Moreover, a small population of CSCs can form the bulk of the tumor mass, highlighting CSC’s cancer initiating potential (Sancho et al., 2015). This theory was further supported via *in vivo* experimentation on squamous skin cancer cells that confirmed the existence of two distinct cell populations: one population had stem cell-like characteristics, and the other population had a slower cycling rate that generated terminally differentiated cells (Driessens et al., 2012). Conversely, another theory states that CSCs arise from spontaneous dedifferentiation. In this theory, tumor progenitor cells undergo tumorigenesis in response to external toxic exposure, which then induces mutations to create CSCs (Nimmakayala et al., 2019). Accordingly, non-CSCs can also revert to CSCs without having to alter their genetics, downstream (Tsuchiya and Shiota, 2021). Other factors contributing to CSC origin include a metabolic shift, cell fusion, or horizontal gene transfer that drive non-CSCs to reprogram into CSCs (Nimmakayala et al., 2019). Eliminating CSCs alone may not be sufficient to stop cancer progression. Since CSCs can spontaneously differentiate from non-CSCs within the tumor, a comprehensive therapy may be more effective by combination of conventional cancer therapy and new CSC-targeted treatment. Next, we will highlight CSCs’ phenotypic markers, which define the tumor-driving cells and stand as therapeutic targets.

These models of cancer origin suggest two approaches in our understanding not only of cancer origin but also of cancer treatment. Conventional cancer treatments are designed to cause tumor shrinkage assessed by the ablation fraction of tumor mass or fractional kill (Palmer et al., 2019). Although the bulk of the tumor, comprised of differentiated or differentiating cells, may show shrinkage, the CSC subpopulation may escape detection by such treatment and may trigger relapse and metastasis. Since CSCs account for a miniscule number of cells within the tumor, such fractional kill index may not reveal the CSC status after conventional chemotherapies. Targeting the tumor-driving CSC directly may prevent tumor relapse (Walcher et al., 2020) while circumventing the need to treat the entire tumor (Miyoshi et al., 2021). Thus, focusing on CSCs as the pivotal origin of cancer may pave the way for innovative views about cancer onset, progression, and its treatment.

### 2.1 Phenotypic markers associated with cancer stem cells

In-depth analysis of the distinct phenotypic markers on CSCs not only allows us to differentiate CSCs’ unique biological properties from its stem cell counterpart but also provides us with key insights into specific CSC-targeted therapies. Specific CSC markers which are upregulated on the surface of CSCs, referred to as the cluster of differentiation (CD), designate them as unique compared to non-tumorigenic stem cells (Kaur et al., 2021). Similarly, CSCs markers also include proteins unique to CSCs and ATP-binding cassette (ABC) efflux transporters (Pattabiraman and Weinberg, 2014). Among these CSC markers that have been widely characterized include: 1) CD44 routinely found in breast cancer, prostate cancer, and gastric cancer, Head and neck squamous cell (HNSCC) (Müller et al., 2020); 2) CD133 detected in glioblastoma, lung cancer, sarcomas, pancreas, and prostate (Pattabiraman and Weinberg, 2014; Müller et al., 2020); 3) CD90 in glioblastoma, breast cancer (Müller et al., 2020); 4) CD117 in glioblastoma, breast cancer, ovarian cancer, lung cancer (Müller et al., 2020); 5) CD29 in breast cancer, colon cancer (Müller et al., 2020); 6) CD47 found on majority of CSC types; engages signal regulatory protein alpha (SIRPα) on macrophages to inhibit their phagocytosis (Müller et al., 2020); 7) aldehyde dehydrogenase 1 (ALDH1) protein that is upregulated in the majority of CSCs (Pattabiraman and Weinberg, 2014); 8) increased expression of lysyl oxidase (LOX) in CSCs is associated with breast cancer (Tsuchiya and Shiota, 2021), and; 9) efflux pumps upregulated in the majority of CSCs, contributes to CSC resistance to conventional chemotherapy (Pattabiraman and Weinberg, 2014).

### 2.2 Cancer stem cells versus stem cells

Similar to CSCs, stem cells (SCs) have self-renewal and differentiation capabilities, and SCs are defined as either embryonic, germinal, or somatic (Kakarala and Wicha, 2007). Embryonic SCs, derived from the inner cell mast of a blastocyte, are totipotent or pluripotent and can generate into any cell type with unlimited replication potential (Kakarala and Wicha, 2007). Germinal SCs, harvested from the germinal layer of the embryo, differentiate into specific organs (Kakarala and Wicha, 2007). Somatic SCs are multipotent and have the capacity to self-renew and differentiate into many types of cells but are limited to a specialized tissue sub-type (Rossi et al., 2020).

Activation of Hh, Wnt, Notch, and TGF-β signaling pathways in CSCs lead to the induction of the embryonic signaling pathway, which is linked to epithelial-to-mesenchymal transition (EMT) activation that ultimately transforms adhesive cells into a mobile phenotype (Takebe et al., 2011). In fact, the embryonic signaling pathway activity is deregulated in various cancers, such as breast, pancreatic, and lung cancers (Takebe et al., 2011). Interestingly, the reverse process of mesenchymal-to-epithelial transition (MET) is also crucial in cancer progression (Yamamoto et al., 2017). *In vitro* studies evidence that both EMT and MET processes, which are governed by the vital proteins Zinc finger E‐box‐binding homeobox 1 (ZEB1) and Zinc finger protein SNAI2 (SLUG), are crucial for cellular metastasis (Yamamoto et al., 2017). The embryonic pathway and EMT process are associated with both CSC’s invasive metastatic potential as well as traditional SC’s plasticity properties (Scheel and Weinberg, 2011). A secondary theory to CSC origin is that CSCs retain the SC’s EMT infrastructure and reactivate the embryonic pathway, rather than curating CSC metastatic properties *de novo* (Scheel and Weinberg, 2011). In other words, SCs can mutate and evolve into a CSCs with EMT potential. The overlap between embryonic pathways in SCs and CSCs highlights the difficulty in targeted therapeutics that must ablate CSCs while preserving vital SCs. Therefore, future CSC research may need to delve into the intricacies of ZEB1 and SLUG proteins to grasp a better understanding of the embryonic pathway within various stem cell subtypes.

CSC-targeted therapeutics should highlight the differences in surface markers expressed on CSCs compared to normal stem cells. Despite our depiction of known CSC phenotypic markers above (see Section 2.1), there is still no sole ubiquitous marker that encompasses all CSC populations. The ability to differentiate CSCs from SCs poses as an overarching challenge, but certain markers are highly expressed among different CSC subpopulations, namely CD44 (Müller et al., 2020), CD133 (Müller et al., 2020), CD90 (Müller et al., 2020), CD47 (Müller et al., 2020), ALDH1 (Pattabiraman and Weinberg, 2014), LOX (Tsuchiya and Shiota, 2021), and efflux pumps (Pattabiraman and Weinberg, 2014). In comparison, embryonic SCs surface markers include CD9, CD24, CD29, CD90, CD117, and CD324 (Zhao et al., 2012). Somatic SCs are further subdivided into hematopoietic SCs, which give rise to blood cells during hematopoiesis, and mesenchymal stromal cells (MSCs), which spawn cells of mesenchymal origin (Rossi et al., 2020). Moreover, CD34, CD38, CD45, CD90, and CD133 are primary clinical marker for hematopoietic SCs, while CD73, CD90, and CD150 are primary markers of MSCs (Tweedell, 2017; Rossi et al., 2020). Since CD90 is depicted as a common CD marker across the majority of stem cell subtypes, it is more prudent to target CD44 and CD133 as targets for CSC directed therapy. In support of this theory, CD44 and CD133 have been the key targets of liver, pancreatic, gastric, breast, and urinary cancer subtypes (Huang et al., 2022). The most streamlined cancer treatment may also aim to target a combination of CD markers with monoclonal antibody therapy. Most notably combination therapy targeting CD133, CD44, and ALDH (aldehyde dehydrogenase) in breast cancer CSCs halted metastatic progression in pre-clinical models (Croker et al., 2009).

It is known that similar signaling pathways in regulating the self-renewal activity are shared in CSCs and SCs. For example, in regards to the Wnt pathway, Wnt5a, an activator of the non-canonical pathway, is involved in the regulation of CSCs and embryonic SCs, but is not activated in somatic SCs (Zhou et al., 2017). Notch signaling is also required both for SC differentiation and CSC generation via Notch regulated transcription factors (Wang et al., 2009). JAK-STAT signaling is also implicated in maintain somatic SCs’ critical homeostatic and regenerative processes, while STAT signaling also defines CSCs’ self-renewal ability (Herrera and Bach, 2019; Yang et al., 2020). TGF-β mostly partakes in tissue repair and maintenance in somatic SCs through Smad3 induction, and together with CSCs, TGF-β initiates various tumor subtypes (Rossi et al., 2020). Hh signaling activation induces stem cell proliferation via increased proliferation of Sox2+ and Sox9+ in adult pituitary stem cells and Hh signaling serves as vital factor in ovarian somatic SCs (Zhang and Kalderon, 2001; Pyczek et al., 2016). In the same token, Hh is linked with development of CSC formation (Takebe et al., 2015). Lastly, VEGF regulates both hematopoietic SC survival and CSC survival through a similar autocrine loop mechanism (Gerber et al., 2002; Mercurio, 2019a; Müller et al., 2020). Therefore, while CSCs and SCs act through similar signaling pathways, including Wnt, TGF-β, Notch, JAK-STAT, Hedgehog, and VEGF, the pathways fail to reach homeostasis in CSCs, contributing to CSC’s chemoresistance (Takebe et al., 2015). Our present review is to gather a better understanding of CSC’s signaling pathways in order to synthesize novel modes of cancer treatment.

## 3 Signaling pathways linked to cancer stem cells

The phenotypic markers and signaling pathways that are involved in CSC activation and maintenance stand as the same pathways that can be targeted for treating cancer. Because CSCs rely on multi-pronged molecular cues, in particular their signaling pathways, for their stemness, directly manipulating these pathways may abrogate their proliferative phenotype, thereby preventing CSC-mediated relapse (Correia et al., 2022). Among these potent signaling pathways that mediate CSCs include Wnt, Notch, TGFβ-SMAD, Hh, JAK-STAT, VEGF, IL-8, granulocyte macrophage colony-stimulating factor (GM-CSF), and bone morphogenic protein (BMP), and below we will discuss their unique therapeutic targets.

A postulated network of signaling pathways associated with CSC activation and maintenance can be deduced, with the overarching concept that CSCs communicate with the TME via paracrine and juxtracrine signaling pathways (Lau et al., 2017). Some major signaling pathways that have been examined to identify the localization of CSCs include: 1) Wnt found in pancreatic, breast, glioma, leukemia, colon, carcinoma and gastric cancer (Sharma et al., 2021); 2) TGFβ-SMAD observed in pancreatic, breast, and glioma (Peng et al., 2022); 3) Notch in breast, ovarian, glioma, pancreatic, and colon cancer (Zhdanovskaya et al., 2021); 4) Hedgehog detected in leukemia, myeloma, pancreatic, breast and glioma (Pattabiraman and Weinberg, 2014; Chen et al., 2021a); 5) JAK-STAT seen in glioblastoma, colon, prostate, and breast cancer (Hu et al., 2021); 6) platelet derived growth factor receptor (PDGFR) apparent in breast cancer (Farooqi and Siddik, 2015); 7) Nanog in glioma, colon, and gastric cancer (Chen et al., 2021a), and; 8) phosphatidylinositol-3-kinase (PI3K) in glioma, colon, and gastric cancer (Tran et al., 2016).

**FIGURE 1:**
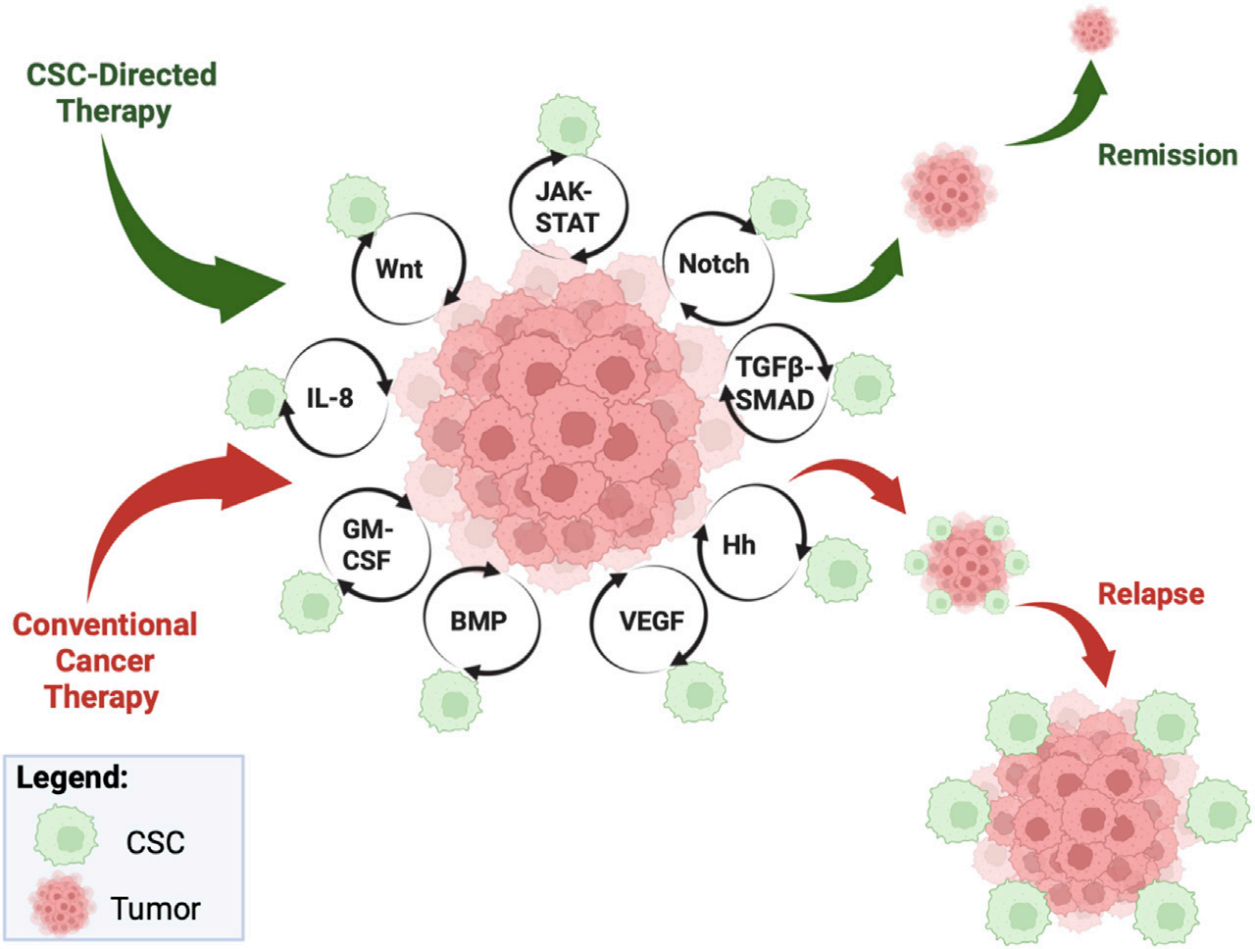
Overview of CSC pathways. Cancer stem cells (CSCs) escape conventional cancer therapy, resulting in an increase in non-CSC progression and maintenance via multi-pronged molecular pathways, such as JAK-STAT, Notch, TGFβ-SMAD, Hh, VEGF, BMP, GM-CSF, IL-8, and Wnt. While conventional cancer therapy will result in relapse (see red pathway), CSC-directed therapy can directly target the CSCs and ultimately afford tumor remission (see green pathway). Figure constructed via Biorender.com.

Based on the stemness feature of CSCs, signaling pathways have been postulated as key mechanisms that participate in CSC activation and maintenance. Principal signaling pathways that have been linked to CSC stemness include: 1) Wnt for CSC formation and maintenance (Kim and Kahn, 2014); 2) Notch for control of CSC replication, survival and differentiation, as well as renewal (Venkatesh et al., 2018); 3) TGFβ-SMAD promotes CSC self-renewal, migration, and invasion of the tumor by facilitating an inflammatory TME (Derynck et al., 2021; Yan et al., 2021); 4) Hh regulates CSC metabolism, thereby increasing CSC generation and maintenance (Statkiewicz et al., 2014), while promoting macrophage recruitment abetting the TME to become more conducive for CSC growth (Lee et al., 2018); 5) JAK-STAT regulates CSC growth, formation, and size (Dolatabadi et al., 2019), and contributes to sustained inflammation of the TME, further exacerbating CSC proliferation (Owen et al., 2019); 6) VEGF facilitate CSCs survival and self-renewal via an angiogenic system to support the cells (Mercurio, 2019b; Tsuchiya and Shiota, 2021); 7) IL-8 facilitates CSC proliferation and expansion by inducing an immunosuppressive TME (David et al., 2016; Kim et al., 2021a; Hirata et al., 2022); 8) GM-CSF contributes to macrophage recruitment to the TME, serving as an immune modulation and hematopoiesis platform for CSC to communicate with tumor cells (Hong, 2016; Liu et al., 2016; Li et al., 2020). Below we will delve into the intricacies of the major CSC signaling pathways (Figure 1).

### 3.1 Wnt signaling pathway in CSCs

#### 3.1.1 Wnt ligand-receptor engagement

Wnts comprise a family of nineteen glycoproteins, which act as ligands (Komiya and Habas, 2008). The Wnt ligand binds to one of the ten types of Frizzled (Fz) extracellular receptors, which is categorized as a seven-transmembrane G-protein coupled receptors (GPCRs) (Komiya and Habas, 2008). There are two main Wnt pathways: 1) the canonical pathway, which is dependent on a low-density-lipoprotein-related protein5/6 (LRP5/6) co-receptor, and 2) the non-canonical β-catenin-independent pathway, which features ROR1/ROR2/RYK co-receptors, which can be further subdivided into the Planar Cell Polarity (Wnt/PCP) and the Wnt/Ca^2+^ pathways (Komiya and Habas, 2008; Ackers and Malgor, 2018). Most notably, the canonical Wnt pathway features a central element, β-catenin, which upon translocating to the nucleus, regulates gene expression by recruiting CREB-binding protein (CBP) to form complexes with the transcription factors, T-cell/lymphoid enhancer (TCF/Lef) (Wiese et al., 2017). If the Wnt ligands fail to activate the receptor, β-catenin is directed to a degradation complex composed of the tumor suppressor Adenomatous Polyposis Coli (APC), AXIN1/2, and kinases CK1α/GSK-3β (Duchartre et al., 2016). Tankyrase (TNKS) is an enzyme that mediates the ubiquitination and degradation of AXIN1/2; most notably tankyrase inhibitors will upregulate the function of the β-catenin degradation complex, serving as a promising target for therapeutics (Wang et al., 2021). Under normal conditions, the Wnt pathway serves a vital role in embryogenesis and the cell cycle, in which β-catenin promotes differentiation and development of specific T-cells, dendritic cells, and tissue systems (Kaldis and Pagano, 2009; Zhan et al., 2017). However, CSCs will “hijack” the Wnt pathway, resulting in uncontrolled proliferation of cancer cells (Fang et al., 2016). Over time, Wnt remains constitutively active due to mutations in tumor suppressor genes and oncogenes in select cancer subtypes (Fang et al., 2016). The large number of Wnt ligand-receptor complexes trigger multiple complex cascades, which play a large role in various types of cancer. For example, Wnt pathway activation results in reduced survival rates in over 50% of breast cancer patients, Wnt signaling modulates gastrointestinal cancers, and the balance between canonical and non-canonical Wnt signaling is linked to melanoma progression (Zhan et al., 2017). However, due to the complexity of the cascade, current Wnt targeted therapeutics are typically halted at the preclinical stage or phase I/II stages of clinical trials (Katoh, 2017). Regardless, further exploration into combined Wnt therapeutics and targeted treatments will provide valuable insight on the intricacies of CSCs.

#### 3.1.2 Wnt upstream and downstream mechanisms

Wnt pathways exhibit crosstalk with the Ras/MAP kinase, PI3/Akt, PLCγ, Notch, Hh and TGFβ/BMP pathways through various feedback loops (Katoh, 2017). Specifically, TGF-β pathway upregulates Wnt2B and Wnt3 expressions in turn activating the canonical Wnt pathway, while Wnt5A and Wnt11 genes are upstream activators of the non-canonical Wnt pathway (Katoh, 2017; Lecarpentier et al., 2019). Both Wnt signaling pathways require activation by the endoplasmic reticulum acyltransferase Porcupine5-7 (PORCN) (Liu et al., 2022). Specifically, PORCN is a membrane-bound O-acyltransferase that is required for the covalent attachment of fatty acids to Wnt ligands in a palmitoylation process, a vital step in Wnt ligand-receptor activation (Guan and Fierke, 2011; Liu et al., 2013a). Upon receptor activation, Dishevelled (Dsh/Dvl) is the first downstream activated intracellular signaling protein, which is involved in each branch of the Wnt cascade (Komiya and Habas, 2008). Activation of the canonical Wnt cascade upregulates the Hh cascade in rodent breast CSCs downstream, while the non-canonical Wnt cascade triggers PI3K-Akt activation promoting CSC survival (Katoh, 2017). Activation of Dsh also leads to β-catenin accumulation in the cytosol, which will then translocate to the nucleus to bind the TCF/Lef to induce cyclin D1, cMYC, monocarboxylate transporter 1 (MCT1), pyruvate dehydrogenase kinase (PDK), and fibronectin target genes. T cell factor 1 (TCF1) is also a downstream transcription factor of canonical Wnt signaling and plays a key role in the development on CD8^+^ memory and effector T cells (Lecarpentier et al., 2019).

#### 3.1.3 Wnt associated genes, markers, and molecules

Many human cancers are associated with mutation or loss-of-function in both canonical and non-canonical Wnt genes: 1) abnormal catenin beta 1 gene (CTNNB1), a downstream co-activator of TCF/Lef, is associated with human breast cancer (van Schie and van Amerongen, 2020) 2) tumor suppressor APC mutations are mainly associated with colorectal cancers (Aghabozorgi et al., 2019), and 3) mutated AXIN1/2 genes, which encode the AXIN protein, a key component of the β-catenin degradation complex, are associated with gastrointestinal cancers (Mazzoni and Fearon, 2014; Zhan et al., 2017; Lecarpentier et al., 2019). The hallmark of canonical Wnt associated cancers is an upregulation of cyclin-D1 and C-Myc genes, which are associated with the cell cycle (Lecarpentier et al., 2019). Interestingly, the mutations in the canonical Wnt pathway are commonly accompanied by mutations in the circadian genes: CLOCK, BMAL1, PER (Lecarpentier et al., 2019). Notable canonical Wnt pathway associated CSC surface markers include: 1) LGR5, a Wnt target gene that encodes the receptor for the R-spondin (RSPO) ligand, which is associated with colorectal, pancreatic, endometrial, and intestinal CSCs (Barker and Clevers, 2010; Lin et al., 2015; Tomita et al., 2020), 2) epithelial cell adhesion molecule (EPCAM) upregulation associated with colon cancer (Zhou et al., 2015), 3) CD44+/CD133+ CSCs are positive regulators of the Wnt pathway associated with prostate (Acikgoz et al., 2021) and colorectal cancer (Tsuchiya and Shiota, 2021), and 5) CD44v6 associated with colorectal CSCs (Lin et al., 2015; Duchartre et al., 2016; Katoh, 2017; Zhan et al., 2017). TERT genes, which maintain CSC’s long telomeres, enhance binding of β-catenin to its promoter, thereby highlighting the correlation between telomerase activity and the Wnt pathway (Zhan et al., 2017). Overall genes associated for the canonical Wnt pathway initiate the EMT of CSCs, while non-canonical Wnt genes are responsible for the persistence and metastasis of CSCs (Katoh, 2017). CSCs undergo EMT to transition from their previous static epithelial cell state to gain migratory and anti-apoptotic abilities (Pattabiraman and Weinberg, 2014). Also of interest, the key genes, AXIN2, APC downregualted-1 gene (APCDD1), and Dickkopf Wnt pathway inhibitor 1 (DKK1) (Koinuma et al., 2006; Sato et al., 2007; de Sousa and Vermeulen, 2016), are negative regulators of the Wnt pathway, so silencing these genes will inhibit CSC expansion. Combined therapeutics should target both the canonical and non-canonical Wnt pathways to irradicate CSCs.

#### 3.1.4 Wnt signaling and cancer: preclinical and clinical studies

The Wnt signaling pathway plays a critical role in stem cell biology and is implicated in CSC/tumor initiating cell population with aberrant Wnt signaling associated with tumor formation, suggesting that arresting Wnt signaling may block CSC maintenance (Kim and Kahn, 2014). Therapeutics which target the Wnt pathway have four general categories: 1) ligand/receptor-targeted drugs (ex. cirmtuzumab, rosmantuzumab and vantictumab): are currently in clinical trials and show anti-CSC effects, 2) PORCN inhibitors are under clinical trials targeting the small molecule PORCN, an O-acyltransferase that is required for Wnt ligand palmitoylation (ex. as IWP-2, WNT974, and ETC-159) (Liu et al., 2013b), 3) Tankyrase inhibitors, such as AZ1366, G007-LK, and JW55, upregulate the destruction complex via AXIN1/2 and are undergoing preclinical testing, 4) β-catenin inhibitors block CSC motility, most notably ICG-001 (and the clinical equivalent PRI-724) are in phase I/II clinical trials (Katoh, 2017; Wang et al., 2021). β-catenin inhibitors are challenging therapeutics due to the complexity of the Wnt pathway, but specific small molecule and monoclonal antibody treatments for select Wnt pathway proteins may also prove successful. For example, ICG-001, a small molecule inhibitor, specifically targets the CBP to arrest tumor growth in both animal and human cancer cell lines (Pattabiraman and Weinberg, 2014; Deng et al., 2020; Wiese et al., 2020). LF3 another small molecule inhibitor of the β-catenin/TCF4 interaction, which was found in an *in vitro* colon cancer mouse cell line, was shown to block CSC’s self-renewal, inhibit migration, and trigger differentiation into a benign tumor state, while not interfering with any other signaling pathway (Fang et al., 2016). Anti-FZD mAb, anti-ROR1 mAb, anti-RSPO3, and anti-LGR5 mAb therapeutics are currently in preclinical or clinical trials (Katoh, 2017). In another study, knockdown of mutated Wnt pathway members in chronic lymphocytic leukemia (CLL) cells showed that leukemia initiating cells (LIC) are dependent on the Wnt pathway for survival (Zhan et al., 2017). Studying patient specific CSC’s for their EMT and MET, immune editing, and metabolism through a genome sequencing process referred to as “omics monitoring” is vital for Wnt targeted therapy (Katoh, 2017). Through omics monitoring, Wnt pathway specific therapeutics can be optimized with combination therapy to target CSCs.

**FIGURE 2:**
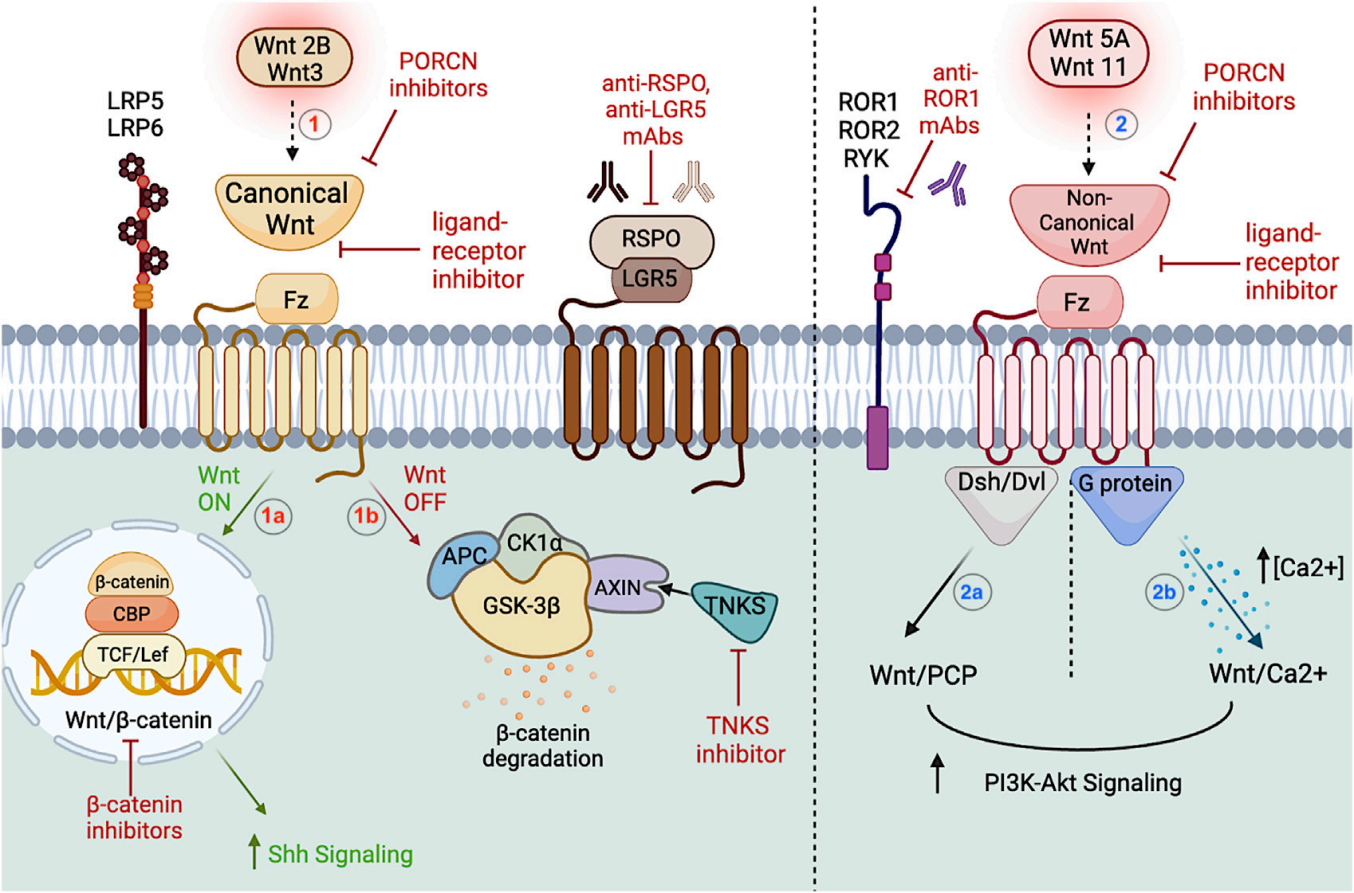
Wnt signaling pathway in CSCs. The left portion of the figure depicts the canonical pathway, which upon Wnt2B and Wnt3 activation, the ligand will bind to the Fz receptor in association with the LRP5/6 co-receptor (1). LGR5 is a membrane bound target GPCR encoding the RSPO protein associated with upregulating CSCs in the canonical pathway. Upon receptor activation (Wnt ON), β-catenin will translocate to the nucleus and form complexes with TCF/Lef to regulate CBP expression (1a). If the Wnt ligand fails to activate the receptor (Wnt OFF), β-catenin is directed to a degradation complex composed of APC, AXIN, and kinases CK1α/GSK-3β (1b). TNKS polymerase upregulates the destruction complex via AXIN1/2. Ultimately, the Wnt/β-catenin results in downstream Hh cascade upregulation. Featured inhibitors of the canonical pathway are PORCN inhibitors, ligand-receptor inhibitors, anti-RSPO and anti-LGR5 mAbs, TNKS inhibitors, and β-catenin inhibitors. The right portion of the figure depicts the non-canonical pathway, which upon Wnt5A and Wnt11 activation, the ligand will bind to the Fz receptor in association with the ROR1/ROR2/RYK co-receptors (2). The non-canonical pathway can be further subdivided into Wnt/PCP (2a) and the Wnt/Ca^2+^ pathways (2b), which upon activation of Dsh/Dvl and G proteins, will upregulate PI3-Akt signaling downstream. Featured inhibitors of the non-canonical pathway are PORCN inhibitors, ligand-receptor inhibitors, and anti-ROR1 mAbs. Figure constructed via Biorender.com.

Despite extensive research on the Wnt signaling pathway, as of March 2023 there is still no FDA approved drug to treat cancer that specifically targets Wnt signaling. Notwithstanding the number of Wnt inhibitors in phase I/II clinical trials [NCT02413853, NCT02278133], overall safety and effectiveness remains a challenge as generalized Wnt inhibitors have detrimental effects on embryogenesis and overall cell homeostasis (Kaldis and Pagano, 2009; Zhan et al., 2017). For instance, the PORCN inhibitor WNT974 (aka LGK974) not only resulted in tumor growth inhibition in head and neck cancer cell lines but also decreased intestinal epithelium when administered at high doses (Liu et al., 2013a). Moreover, in human clinical trials, WNT974 treatment was generally well tolerated to treat advanced solid tumors with the most common side effect being dysgeusia, altered taste perception, in 50% of patients [NCT01351103], (Rodon et al., 2021). However, higher grade adverse events are expected to occur in clinical trials involving OMP-54F28, a Wnt recombinant fusion protein, as bone density loss had to be supplemented with vitamin D3 and calcium carbonate [NCT01608867]. Overall, concerns regarding Wnt pathway inhibition include effects on intestinal cells, bone density, dysgeusia, decreased appetite, fatigue, muscle spasms, and overall cell homeostasis (Jimeno et al., 2017). The questions as to whether the benefit of Wnt inhibitors outweighs the risks remains. However, despite diverging opinions regarding Wnt targeted signaling in cancer, as our understanding of the Wnt pathway increases, so does the growing enthusiasm for novel Wnt therapeutics (Figure 2).

### 3.2 Notch signaling pathway in CSCs

#### 3.2.1 Notch ligand-receptor engagement

The Notch signaling pathway not only plays a critical role in embryogenesis, but also in regulating CSC proliferation, maintenance, and differentiation, with notable contribution to angiogenesis (Venkatesh et al., 2018). Classically, Notch serves a dual role acting as both a tumor suppressor and an oncogene, at times leading to tumorigenesis depending on tissue type or genetic mutation (Meisel et al., 2020). Depending on the microenvironment, Notch is typically downregulated in prostate, skin, lung, liver, and some breast cancers, while Notch is upregulated in gastric, colon, pancreatic, and some breast cancers (Yang et al., 2020). Under hypoxic conditions, the Notch pathway undergoes EMT, therefore the consensus is that CSCs typically upregulate the Notch pathway to enhance their stemness properties (Meisel et al., 2020). Notch signaling initiates when Delta/Serrate/Lag2 (DSL) transmembrane Notch ligands (i.e., Delta-like 1/3/4, or Jagged 1/2) bind to a Notch receptor (Notch1–4) in a juxtracrine fashion, triggering cleavage via ADAM-10 protease, followed by gamma-secretase (γ-secretase) intramembrane protease, which releases the intracellular Notch portion from the plasma membrane (Anders et al., 2006; Venkatesh et al., 2018). This cleavage results in Notch’s intracellular domain (NICD) translocating to the nucleus and binding to a CBF1/Suppressor of Hairless/Lag-1 (CSL) transcription factor, ultimately activating target genes (Venkatesh et al., 2018; Yang et al., 2020). Since activation of the Notch signal transduction pathway in CSCs, treatment directed at this pathway may control CSC replication, survival, and differentiation, thus regulating CSC renewal and modulating CSC-mediated tumor formation and its recurrence (Venkatesh et al., 2018).

#### 3.2.2 Notch upstream and downstream mechanisms

The following upstream activators of the Notch pathway contribute to the self-renewal capacity of CSCs: 1) nitric oxide (NO) synthase increases the stemness of liver CSCs, and NO targeted therapy increases tamoxifen potency in breast CSCs (López-Sánchez et al., 2021), and 2) MAP17, a 17 kDa non-glycosylated membrane protein that is associated with an increase in reactive oxygen species (ROS), is upregulated in cervical, breast, colon, and lung CSCs (Guijarro et al., 2007; Garcia-Heredia et al., 2017; Yang et al., 2020). Hyperactivation of Notch can typically occur through a genetic mutation resulting in either increased expression of the cleaved portion of Notch’s intracellular domain, or ligand-independent receptor activation (Meisel et al., 2020). However, genetically independent receptor activation via ligand abundance or increased ligand-receptor affinity could also result in carcinogenesis (Meisel et al., 2020). For example, an increase in the expression of Notch2 and Jag1 are correlated with an increased incidence of medulloblastoma and prostate cancer, respectively (Meisel et al., 2020). Downstream, Notch communicates with a variety of immune cells in the TME, such as myeloid derived suppressor cells (MDSCs), tumor associated macrophages (TAMs), and T regulatory cells (Tregs) (Venkatesh et al., 2018). MDSCs are exceedingly dynamic cells that further promote CSC stemness via NO secretion via both the STAT-3 (Müller et al., 2020) and Notch pathway (Chen et al., 2021a). Notch signaling also regulates the downstream proto-oncogene C-Myc, cell cycle regulators (ex. cyclin, D1, and Cdkn1) and can act as a positive or negative regulator on itself via Deltex E3 Ubiquitin Ligase 1 (DTX1), a protein coding gene (Matsuno et al., 1995; Meisel et al., 2020). The Hh pathway also induces C-myc expression, which acts in parallel with the Notch pathway (Meisel et al., 2020). The Notch pathway also exhibits crosstalk with STAT3 signaling to promote EMT transition (Shan et al., 2021).

#### 3.2.3 Notch associated genes, markers, and molecules

The Notch signaling pathway significantly contributes to CSC maintenance in several cancer types, paving the way for development of Notch inhibitors as an anti-cancer strategy (Purow, 2009). The following markers and molecules are known to enhance and maintain the stemness properties of CSCs via Notch signaling: 1) delta-like ligand 4 (DLL4) maintains gastric CSCs and regulates tumorigenesis (Segami et al., 2021), 2) tumor necrosis factor-α (TNFα) inhibition in liver CSCs decreases cancer metastasis (Renz et al., 2018), 3) BMP4 has a dual role - it is upregulated in breast CSCs (Bach et al., 2018) while BMP4 inhibited hepatic CSC self-renewal (Zhang et al., 2012a), 4) Jagged 2 is upregulated on breast and lung CSCs under hypoxic conditions, and 5) blocking VEGFR2 causes skin CSC pool size reduction (Beck et al., 2011; Takebe et al., 2014; Yang et al., 2020). Similarly, the following genes aid in CSC expression via Notch: 1) Gli3 in oral squamous cell carcinoma (SCC) CSCs, 2) Notch1 in ovarian CSCs under hypoxic conditions, 3) BRCA1 in breast CSCs, 4) Hairy enhancer of split genes (Hes1-7) in medulloblastoma CSCs (de Antonellis et al., 2013), 5) Hey (Hey1, Hey2 and HeyL) in HNSCC (Moon et al., 2019), 6) Notch-regulated ankyrin-repeat protein (NRARP) associates with non-small lung cancer (Liao et al., 2018), 7) cyclin D1 silencing suppresses liver CSC differentiation (Zhang, 2020), 8) DVL1 gene is upregulated on glioblastoma CSCs (Hsu et al., 2021), and 9) ADAM19 is elevated on breast CSCs (Takebe et al., 2014; Meisel et al., 2020; Yang et al., 2020). In a microarray study, the following genes: Notch1, Hes4/5, Hey1/L, and NRARP were inversely proportional to concentrations of the Notch inhibitors (i.e., MK-0752), confirming their effectiveness as biomarkers (Takebe et al., 2014). Strategies that target Notch genes, such as Notch 1, may prove beneficial for cancer treatment (Takebe et al., 2015; Gharaibeh et al., 2020). The following molecules are known to inhibit Notch signaling: 1) microRNA-34a is a potent tumor suppressor known to inhibit gastric CSCs (Jang et al., 2016), 2) PER3 polymorphisms are associated with colorectal CSCs, 3) miR-200b-3p expression is associated with pancreatic CSCs and downregulates colorectal cancer (CRC) cells (Feifei et al., 2019), and 4) miR-26a expression is found on osteosarcoma CSCs and inhibits ovarian (Gao et al., 2020) and colorectal cancers (Yang et al., 2020; Chen et al., 2021b). Inhibition of these Notch signaling molecules could also serve as candidate targets for developing potential cancer therapeutics (Zhang et al., 2017a; Zhang et al., 2017b).

#### 3.2.4 Notch signaling and cancer: preclinical and clinical studies

The overall premise in targeting Notch signaling pathway is that combining a specific notch inhibitor with a traditional chemotherapeutic will result in a more potent anti-cancer treatment plan. For example, Notch1 inhibition in combination with a chemotherapeutic drug reduced CSC self-renewal in HNSCC CSCs *in vitro* and *in vivo*, confirming Notch1 as an ideal target for cancer treatments (Fukusumi and Califano, 2018; Venkatesh et al., 2018)*.* Clinical agents which target Notch signaling fall under two categories: 1) γ-secretase inhibitors (GSIs) (ex. peptide isosteres, azepines, and sulfonamides) inhibit cleavage of Notch resulting in suppression of angiogenesis and apoptosis of tumor cells, and 2) monoclonal antibodies (mABs) that interfere with Notch ligand-receptor bonding or prevent the conformational change required for cleavage (Venkatesh et al., 2018; Yang et al., 2020). The following GSIs have emerged as promising therapeutics for inhibiting CSCs specifically by modulating the Notch pathway: 1) RO4929097 combined with bevacizumab decreased glioma CSCs in phase I clinical trials, and other RO4929097 combinations are known to combat advanced solid tumors [ NCT0113123], 2) PF-03084014 combined with gemcitabine decreased pancreatic CSCs *in vivo* and decreased desmoid tumors in phase II studies, 3) MRK003 decreased glioma CSCs *in vivo,* and 4) MK-0752 combined with docetaxel decreased metastatic breast cancer CSCs *in vitro*, MK-0752 combined with cisplatin treated ovarian cancer in vivo, MK-0752 combined with gemcitabine treated ductal adenocarcinoma of the pancreas in preclinical trials, and MK-0752 showed inhibition of pediatric central nervous system tumors in phase I clinical trials (Venkatesh et al., 2018; Yang et al., 2020)*.* Interestingly, GSIs proved ineffective against in triple negative (ER, PgR and HER-2) breast cancer, which displayed elevated Notch signaling, emphasizing the unique complexity of CSCs and tumor types (Meisel et al., 2020).

In parallel, preclinical studies and clinical trials which feature mAbs that target the Notch pathway and inhibit CSCs have also shown encouraging results: 1) Tarextumab (OMP-59R5) decreased breast, small-cell lung, ovarian, and pancreatic CSCs by 40% after chemotherapy termination *in vivo* and effectively decreased solid tumors in phase Ib/II clinical trials by targeting Notch2/3 [NCT01277146], and 2) enoticumab (ex. REGN421, SAR153192) partially treated ovarian cancer and solid tumors by inhibiting DLL4 (Chiorean et al., 2015; Venkatesh et al., 2018; Smith et al., 2019) [NCT0087155]. Interestingly, through a combination of both a GSI and a mAb in early-stage cancers, there is a marked increase in chemotherapeutic effect, but a particular side-effect that commonly arises is gastrointestinal toxicities (Takebe et al., 2014; Venkatesh et al., 2018). Through identification of patient-specific Notch pathway pharmacodynamic biomarkers, the patient can be matched to the appropriate Notch inhibitor to create an appropriate treatment plan (Takebe et al., 2014; Venkatesh et al., 2018).

**FIGURE 3:**
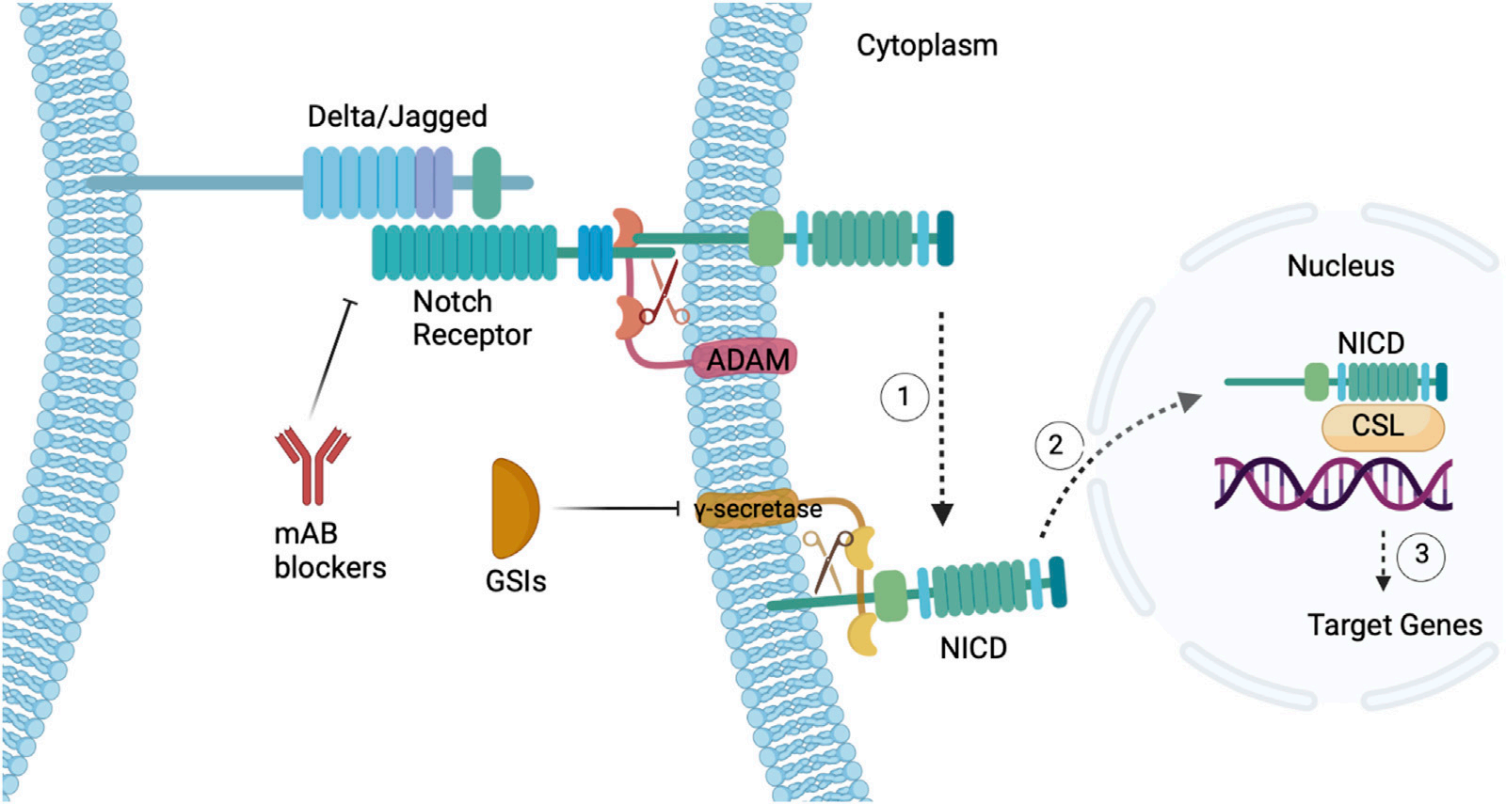
Notch signaling pathway in CSCs. (1) Delta/Jagged are notch ligands that bind to a Notch receptor in a juxtacrine fashion triggering cleavage via ADAM protease and γ-secretase. (2) Upon cleavage, the NICD translocates to the nucleus and binds to the CSL transcription factor, (3) ultimately activating target genes. Featured inhibitors of the Notch pathway are mAbs that target the ligand-receptor interaction and GSIs that target γ-secretase. Figure constructed via Biorender.com.

The discovery of effective, safe Notch inhibitors that predict a positive clinical outcome is essential. However, the Notch signaling pathway is not only vital for CSCs self-renewal but is also necessary for embryogenesis and angiogenesis (Venkatesh et al., 2018), posing as a hurdle for selective Notch-targeted therapies. Research is currently focused on Notch-selective GSIs, which inhibit angiogenesis and trigger tumor cell apoptosis, as well as inhibitory monoclonal antibodies that target Notch pathway molecules (Venkatesh et al., 2018). For example, in an *in vivo* study Notch pathway inhibition via delivery of a GSI reduced the percentage of CSCs within the tumor, while activation of the Notch pathway increased the percentage of CSCs within the tumor (Abel et al., 2014). Moreover, in a phase I clinical trial of Tarextumab, a mAb Notch2/3 inhibitor that targets solid tumors, GI toxicity was the most common adverse event, with patients experiencing diarrhea (81%), fatigue (48%), nausea (45%), anorexia (38%), vomiting (38%), abdominal pain (24%), and constipation (24%) [NCT01277146] (Smith et al., 2019). Tarextumab was also tolerated better at low doses <2.5 mg weekly [NCT01277146] (Smith et al., 2019). Despite intestinal toxicity being the most common side effect of Notch inhibitors, *in vivo* testing confirms that GI toxicity can be reduced by implementing a Notch3-selective antibody rather than Notch1/2 inhibitors (Wu et al., 2010). Therefore, in order to limit the toxicity of GSIs and mAbs doses must be selective, moderated, and taken intermittently. In conclusion, while drugs targeting the Notch pathway are typically halted at phase I/II clinical trials, an in-depth evaluation of Notch signaling has paved the way for novel CSC therapeutics with the ultimate goal of curing cancer (Figure 3).

### 3.3 JAK-STAT signaling pathway in CSCs

#### 3.3.1 JAK-STAT ligand-receptor engagement

The JAK-STAT signaling pathway is integral to the survival, self-renewal, maintenance, and metastasis of CSCs (Yang et al., 2020). The binding of ligands, typically cytokines or interferons (IFNs), to the cytokine cell-surface receptor results in receptor dimerization, which induces both positive and negative regulatory pathways (Kisseleva et al., 2002). After receptor-ligand engagement, the tyrosine kinase JAK (i.e., JAK1-3, and Tyk2), which is composed of seven domains, will phosphorylate the receptor, creating binding sites for proteins possessing an SH2 domain, such as STAT proteins (Kisseleva et al., 2002; Yang et al., 2020). STAT proteins (i.e., STAT1-4, STAT5a/b, STAT6) are transcription activators composed of N and C terminals, a DNA-binding region, and SH2/3 domains (Yang et al., 2020). Upon STAT binding to the receptor, it also undergoes tyrosine phosphorylation, causing the STAT to dissociate from its corresponding receptor, form an anti-parallel dimer, and translocate to the nucleus to affect target downstream molecules (Schindler et al., 2007; Yang et al., 2020).

#### 3.3.2 JAK-STAT upstream and downstream mechanisms

Specifically, JAK-STAT pathway constitutive activation and mutation are associated with many tumors (Yang et al., 2020). The following JAK-STAT upstream activators contribute to the self-renewal capacity of CSCs: 1) IL-6 activates the JAK1/STAT3 pathway in endometrial CSCs, induces EMT in breast and colorectal CSCs (Zhang et al., 2018a), 2) IL-10, an immunosuppressive cytokine, induces stemness properties in lung CSCs (Yang et al., 2019), 3) GM-CSF will create a positive feedback loop between CSCs and TAMs within the TME (Kokubu et al., 2016), 4) PDGF increased stemness and metastatic potential in ovarian CSCs (Raghavan et al., 2020). Downstream, JAK-STAT upregulates PI3-Akt signaling and promotes activation of the MAPK/ERK pathway. The PI3K protein (similar to STAT) contains an SH2 domain, so it can bind to JAK phosphorylated tyrosine receptors and activate the PI3-Akt pathway (Rawlings et al., 2004). In a similar fashion Grb2, an integral protein for MAPK/ERK signaling, also features a SH2 domain, which allows it to bind JAK phosphorylated receptors, and activate the MAPK/ERK pathway (Rawlings et al., 2004). JAK-STAT signaling can integrate multiple signaling pathways, contributing to its complexity and therapeutic potential.

#### 3.3.3 JAK-STAT-associated genes, markers, and molecules

JAK-STAT signaling behaves as a double-edged sword, whereby STAT3 is commonly linked to CSC’s immunosuppression capacity and aberrant TME, while STAT 1/2 activates an anti-tumor immune response via interferons, (i.e., IFN I/II) (Owen et al., 2019; Yang et al., 2020). Therefore, therapeutic avenues should navigate the complexity of JAK-STAT signaling with caution. The following genes, markers, and molecules are known to enrich the stemness properties of CSCs via JAK-STAT signaling: 1) Oct4 promotes ovarian CSCs, and lung CSCs through M2 macrophage polarization (Lu et al., 2020); 2) erythropoietin (Epo) upregulates breast and colorectal CSCs, as well as human gliomas via Epo-dependent constitutive activation of STAT-5 (Kondyli et al., 2010); 3) retinol-binding protein 4 (RBP4) promotes colon CSCs self-renewal via the JAK2-STAT3 pathway (Karunanithi et al., 2017); 4) Hypoxia inducible factor-1 alpha (HIF-1α), a key transcription factor in cancer progression (Rashid et al., 2021), is associated with glioma CSC upregulation (Yang et al., 2020); 5) miR-500a-3p in hepatocellular carcinoma CSCs leads to STAT3 constitutive activation (Yang et al., 2020); 6) TH17, an immunosuppressive cytokine, upregulates CSCs via the secretion of IL-17 via a STAT3-dependent signaling pathway in ovarian, colorectal, and gastric cancers (Chen et al., 2021a). In addition to STAT3, the MAPK (Mitogen Activated Protein Kinase) signaling pathway appears to also solicit the link between TH17 and CSCs in ovarian and pancreatic cancers (Chen et al., 2021a). The following molecules are known to inhibit JAK-STAT signaling: 1) Mir-218 inhibits the JAK-STAT3 pathway resulting in downregulation of lung CSC’s self-renewal capacity (Yang et al., 2017); 2) Ajuba, a LIM domain-containing scaffolding protein, promotes the proliferation of colorectal CSCs through suppression of JAK1/STAT1 (Jia et al., 2017); 3) Von Hippel–Lindau (VHL) cell surface proteins act via the JAK2/STAT3 pathway to suppresses self-renewal ability of glioma CSCs (Yang et al., 2020). Recognizing the dual properties of JAK-STAT, the activation and inhibition of these JAK-STAT signaling regulators represent potential candidate targets for cancer therapy.

#### 3.3.4 JAK-STAT signaling and cancer: preclinical and clinical studies

Due to the integral contribution of the JAK-STAT signaling pathway in CSC preservation, JAK-STAT inhibitors serve as a notable approach to anti-cancer treatment. Targeted JAK-STAT pathway therapeutics fall into one of three categories: 1) cytokine or receptor antibodies, 2) JAK inhibitors, 3) STAT inhibitors (Hu et al., 2021).

In reference to the first category, antibody-cytokine novel fusion proteins (immunocytokines) exert an anti-cancer effect, as shown in reference to IL-2 in phase I/II clinical trials (Mortara et al., 2018). Ruxolitinib in combination tocilizumab, antibodies against IL-6, show improved survival ovarian cancer tumors *in vivo* (Qureshy et al., 2020). Ruxolitinib enhances cancer treatment in HNSCC, pancreatic, and glioblastoma (Qureshy et al., 2020).

Under the second category, JAK inhibitors typically result in immunosuppressive effects through decreasing proinflammatory cytokines. For example, pacritinib is a JAK2 inhibitor currently under clinical trials to treat AML, prostate, colon, rectal, and non-small cell lung cancer, but no response was observed in colorectal cancer patients (Hu et al., 2021). Cerdulatinib, a JAK1/2 inhibitor, is undergoing clinical trials for treatment of non-Hodgkin’s lymphoma [NCT04757259].

In the third category, STAT inhibitors include SMIs, peptide inhibitors, STAT-targeting small interfering RNAs (siRNA), ASOs that interfere with STAT mRNA, and decoy oligonucleotides (ODNs) (Hu et al., 2021). STAT3 inhibition can occur via the SMI drug, Napabucasin (Chen et al., 2021a). Napabucasin suppresses MDSC immunosuppressive capacity in melanoma-bearing mice (Bitsch et al., 2022) and is in phase I clinical trials for assessment of safety, tolerability, and pharmacokinetics in healthy volunteers (Dai et al., 2021) and patients with metastatic colorectal cancer (Taniguchi et al., 2021). A notable peptide inhibitor is PY*LKTK, which was shown to disrupt STAT3:STAT3 dimerization *in vitro* (Yue and Turkson, 2009). siRNA targeting STAT 5b enhances the chemosensitivity of gastric cancer cells to gefitinib in clinical trials (Sun et al., 2015). AZD9150, a STAT3 ASO, is undergoing phase 1 clinical trials for patients with advanced hepatocellular carcinoma (HCC) [NCT01839604] as well as advanced solid malignancies [NCT03394144]. ODNs bind to the DNA binding domain preventing STAT proteins from reacting with their appropriate DNA response element (Furqan et al., 2013). Specific ODN targeting STAT3/STAT5 (ex. K562, U251, A172, etc.) were studied *in vitro* and *in vivo* on a mice xenograft lung cancer model (Furqan et al., 2013).

**FIGURE 4:**
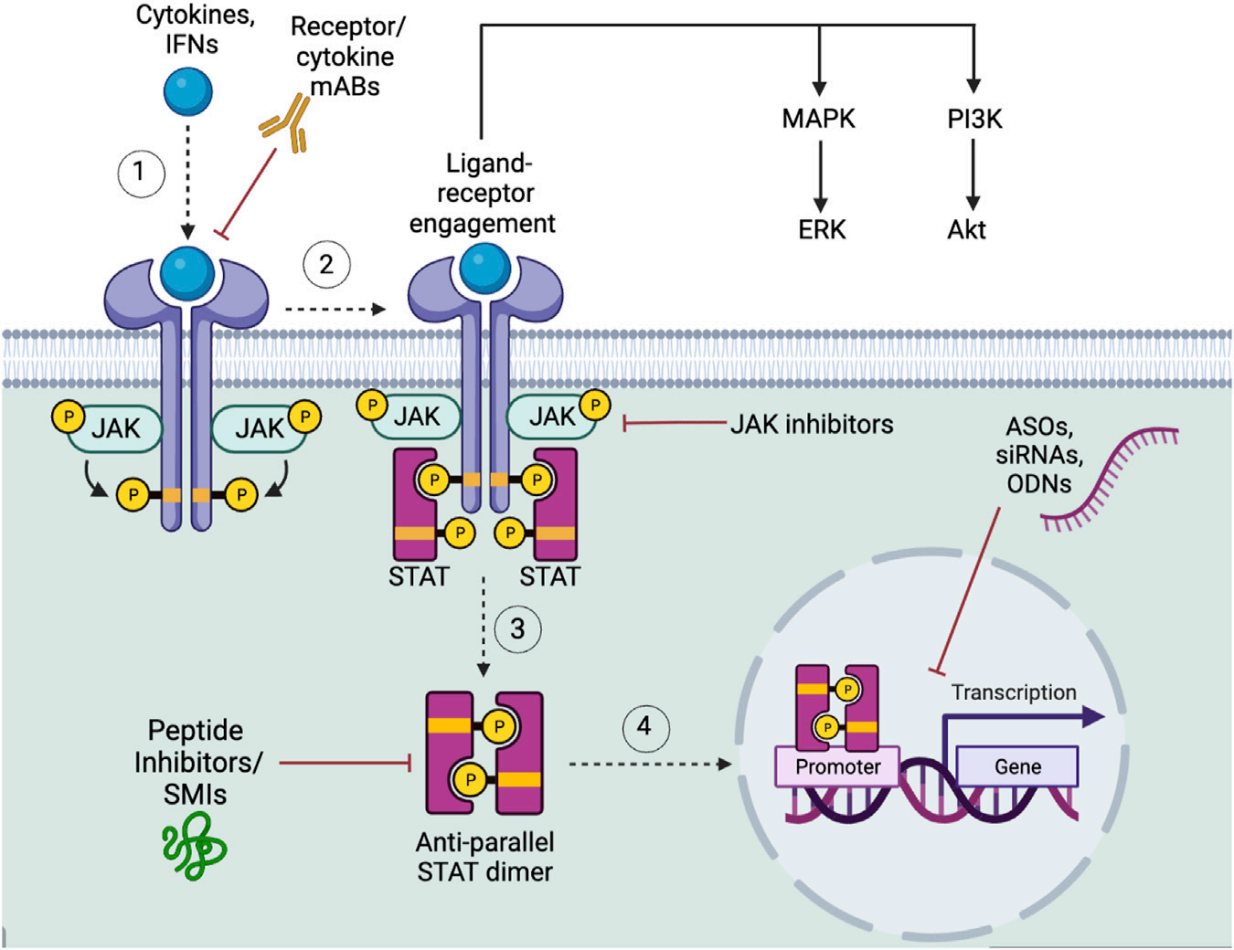
JAK-STAT signaling pathway in CSCs. (1) Upon the activation of the JAK-STAT receptor by the appropriate cytokines or IFNs (2) the tyrosine kinase JAK (i.e. JAK1-3, and Tyk2), will phosphorylate the receptor, followed by STAT proteins (i.e. STAT1-4, STAT5a/b, STAT6) tyrosine phosphorylation, (3) leading to STAT dissociation and formation of its anti-parallel dimer. (4) The STAT dimer will then translocate to the nucleus to affect downstream target molecules. Featured inhibitors of the JAK-STAT signaling pathway include receptor mAb inhibitors, targeted upstream cytokine mAb inhibitors, JAK inhibitors, and STAT inhibitors. STAT inhibitors can be subcategorized into SMIs, peptide inhibitors, siRNAs, ASOs, and ODNs. Downstream, JAK-STAT ligand-receptor engagement activates the MAPK/ERK and PI3K/Akt pathways. Figure constructed via Biorender.com.

JAK-STAT pathway dysregulation is a major contributor to cancer progression. Within the TME, JAK-STAT signaling controls cytokine secretion, inflammatory cascades, and regulates CSCs maintenance and proliferation via upstream and downstream mechanisms. In most cancer subtypes JAK-STAT pathway inhibition serves as a potential chemotherapeutic treatment, but selective targeting remains a challenge due to variabilities in patient genetics, epigenetics, and variations in tumor subtypes. Therefore, JAK-STAT pathway combined therapy targeted drugs should not only inhibit overactivation of the pathway but also delve into the intricacies of JAK-STAT pathway crosstalk as a potential means for novel therapeutics. Current JAK-STAT pathway therapeutics, which include antibody therapy, JAK-inhibitors, and STAT-inhibitors, are typically in phase I/II clinical trials and are focused on administering the drug alone or in combination with chemotherapy, rather than targeting multiple major CSC signaling pathways [NCT03421353, NCT04021082]. JAK-STAT is a complex, non-linear signaling pathway and in some cases crosstalk with JAK-STAT and downstream signaling pathways, such as MAPK/Erk and PI3K/Akt, can contribute to chemotherapy resistance. For example, in a colon cancer cell line, IL-6 secretion mediated the activation of JAK-STAT3 and MAPK/Erk pathways, which increased anti-apoptotic proteins, Bad and Bcl-2, thus leading to chemoresistance to 5-Fluorouracil (Li et al., 2018a). Moreover, JAK1/2 inhibitors, namely AZD1480 for the treatment of solid tumors and Momelotinib that treats non-small cell lung cancer, resulted in clinical trial termination due to neurotoxicity and neutropenia, respectively [NCT01112397, NCT02206763]. Conversely, Ruxolitinib showed improved overall survival rates in a subgroup of pancreatic ductal adenocarcinoma patients with inflammation [NCT01423604]. Despite a clinical trial depicting the benefit of Ruxolitinib in a select patient population, the majority of clinical trials testing the effects of Ruxolitinib resulted in termination, perhaps due to JAK inhibition resulting in decreased immune response, which hinders overall anti-tumor effects [NCT02117479, NCT02119663, NCT01562873], (Schwartz et al., 2017). Despite potential drawbacks in research, JAK-STAT targeted CSC therapy is a novel research avenue with the future direction of curating personalized medicine treatment plans based on variations in JAK-STAT signaling among different tumor subtypes (Figure 4).

### 3.4 Hh signaling pathway in CSCs

#### 3.4.1 Hh ligand-receptor engagement

The hedgehog family is comprised of three homologs: Sonic hedgehog (Shh), Indian hedgehog (Ihh), and Desert hedgehog (Dhh), of which Shh is the best understood (Sasai et al., 2019). Under normal conditions, the hedgehog signaling pathway plays a unique role in embryonic development and cell differentiation (Yang et al., 2020). However, abnormal Hh pathway activation is linked to a variety of cancer types (Schulenburg et al., 2015), and CSCs uniquely express high levels of Hh activation (Yang et al., 2020). When extracellular Hh ligands bind to its downstream twelve-pass transmembrane receptor, Patched (PTCH), such engagement triggers signal transduction, most notably activating downstream GLI transcription factors (i.e., Gli1/2/3) (Pietrobono et al., 2019; Yang et al., 2020). In the absence of ligand-receptor activation, PTCH acts a negative regulator that will inhibit the seven-pass transmembrane GPCR Smoothened (SMO), preventing signal transduction (Pietrobono et al., 2019; Yang et al., 2020). Similarly, in the absence of a ligand, suppressor of fused (SUFU) binds to GLIs, anchors them in the cytoplasm, and prevents them from activating GLI target genes, thereby inhibiting Hh signaling (Rimkus et al., 2016). Selective inhibition of Hh signaling is an effective strategy for impeding cancer progression and halting CSC metastasis.

#### 3.4.2 Hh upstream and downstream mechanisms

The following Hh upstream activators contribute to the maintenance, self-renewal, and regenerative capacity of CSCs: 1) IL-6 activates non-small-cell lung CSCs (Wei, 2019), 2) Gli1 initiates Hh transcription and notably promotes EMT and metastasis of non-small cell lung carcinoma (Jiang et al., 2020), and 3) Gli2 mainly inhibits transcription but in some instances also triggers Hh transcription due to variation in levels of phosphorylation (Pietrobono et al., 2019; Yang et al., 2020). Downstream, the Hh pathway also activates Gli3, a known transcription inhibitor, and Gli3 mutation has been linked to pancreatic adenocarcinoma (Yang et al., 2020). After translation of the Hh protein, the membrane-bound Hedgehog acyltransferase (Hhat) catalyzes a Hh-palmitate linkage and recycles the Hh ligand, so it may again bind to PTCH1 (Rimkus et al., 2016). Hh signaling also contributes to the metastasis of CSCs through upregulation of the following downstream markers: 1) ALDH1 a biomarker associated with early-stage non-small cell lung cancer via Hh signaling (Raz et al., 2012), 2) CD44 a biomarker associated with hepatocellular carcinoma via Hh signaling (Wang et al., 2020), 3) Twist1, Snail, and ZEB1 contribute to CSCs EMT capacity (Rimkus et al., 2016; Li et al., 2021), 4) C-Myc and cyclin-D1 are transcription factors involved in CSC proliferation and cell cycle progression (Rimkus et al., 2016; Elbadawy et al., 2019), 5) Nanog, Oct4, and Sox2 are transcription factors that contribute to CSC self-renewal (Shan et al., 2012; Yin et al., 2015; Rimkus et al., 2016; Yang et al., 2020) 6) PDGFR upregulation on glioblastoma CSCs (Schulenburg et al., 2015; Yang et al., 2020). The Hh pathway also displays crosstalk with the Wnt and Notch pathways via Hh upregulation of Wnt2 and Jagged1, respectively (Ding and Wang, 2017; Yang et al., 2020).

#### 3.4.3 Hh-associated genes, markers, and molecules

Increased Hh signaling is observed in CSCs to support their maintenance, growth, and metastasis (Yang et al., 2020). The following genes, markers, and molecules are known to enrich the stemness properties of CSCs via Hh signaling: 1) CK2α, a protein kinase, supports lung CSCs via upregulation of Gli1 (Zhang et al., 2012b), 2) retinoic acid receptor α2 (RARα2) upregulates Hh and Wnt pathways to maintain myeloma CSCs (Yang et al., 2013), 3) PPM1D, a protein phosphatase, increased Hh signaling via target protein Gli1 in medulloblastoma CSCs (Wen et al., 2016), and 4) lncHDAC2 promotes liver CSCs through Hh signaling (Yang et al., 2020; Jahangiri et al., 2021). Speckle-type POZ (SPOP) is a unique protein in that it at times inhibits Hh signaling in gastric cancer through increasing the degradation of Gli2 (Zeng et al., 2014), while SPOP can also upregulate Hh signaling, decreasing CRC CSC’s rate of apoptosis (Zhi et al., 2016). Similarly, Vasohibin 2 (VASH2) protein suppresses pancreatic CSCs through decreasing SMO and Gli1/2 (Yang et al., 2020), while also functioning as a tumor promoter, stimulating resistance to doxorubicin (DOX) (Mirzaei et al., 2021) upregulating pancreatic CSCs via the Hh and Notch pathways (Liang et al., 2021a). The following molecules are known to inhibit Hh signaling thereby hindering the metastasis of CSCs: 1) BCL6, a transcriptional repressor, inhibits the positive Hh effectors, Gli1 and Gli2, in medulloblastoma CSCs (Yang et al., 2020), 2) RUNX3, a transcription factor, suppresses colorectal CSCs metastasis by blocking Gli1 (Kim et al., 2020), 3) miR-361-3p hinders retinoblastoma CSC self-renewal, by Gli1/3 inhibition and regulates liver CSCs through directly targeting SOX1 (Pietrobono et al., 2019; Qu et al., 2021), 4) miR-326, a scaffolding protein, impedes Gli2 and regulates SMO, thus creating a negative feedback loop for modulating Hh signal transduction (Jiang et al., 2014; Pietrobono et al., 2019), 5) β-arrestin1 (Arrb1), a scaffolding protein, acts as another negative modulator of Hh signaling through acetylation of Gli1, specifically identified in relation to medulloblastoma CSCs (Miele et al., 2017). Probing Hh-associated genes, markers, and molecules may reveal novel pathways or refine existing signaling pathways associated with CSC metastasis, cancer onset, and progression, paving the way for Hh-based cancer therapeutics.

#### 3.4.4 Hh signaling and cancer: preclinical and clinical studies

The following molecules and proteins have emerged as promising therapeutics for inhibiting CSCs by controlling the Hh pathway. Targeted Hh pathway therapeutics fall into one of three categories: 1) SMO inhibitors, 2) Gli inhibitors, and 3) ligand/enzyme inhibitors (Rimkus et al., 2016).

Under the first category, SMO inhibitors prevent Gli activation downstream, leading to target gene inhibition and CSC suppression (Rimkus et al., 2016). LDE225 (also known as Erismodegib/Sonidegib) is a SMO inhibitor as evidenced *in vitro* and *in vivo* as a SMO antagonist (D’Amato et al., 2014), and is currently marketed as Odomzo® to treat advanced stage basal cell carcinoma (BCC) [NCT04066504]. The same SMO inhibitor, Sonidegib, also acts synergistically with JAK2 inhibitors (ex. ruxolitinib) to inhibit HER2-positive triple-negative breast CSCs *in vitro* (Doheny et al., 2020). In the same token, the Hh antagonist Vismodegib, a cyclopamine derivative that binds SMO preventing Gli activation and macrophage recruitment modifier, is a clinically approved therapy to treat BCC and is undergoing phase II clinical trials for medulloblastoma and other cancers (Gampala et al., 2021) [NCT00833417]. Another SMO inhibitor, steroidal alkaloid cyclopamine, is a natural plant product that directly antagonizes Hh *in vivo* (Incardona et al., 1998; Tremblay et al., 2009) and is currently under phase II clinical trials for medulloblastoma [ NCT01878617]. Due to the integral role of SMO in Hh signaling, it is the main target of many therapeutics.

Under the second category, Gli antagonists (GANTs) act downstream of the Hh signaling pathway and, therefore can be activated via Hh-independent pathways (Rimkus et al., 2016). Arsenic trioxide (ATO), a Gli1/2 suppressor, is the first FDA approved drug that targeted the Hh pathway to treat acute promyelocytic leukemia ATO and currently undergoing preclinical testing for a variety of cancers, including prostate and colon cancer (Rimkus et al., 2016; Carpenter and Ray, 2019). GANT-61 inhibits both Gli1/2 downstream effectors and decreases tumor growth *in vivo* and *in vitro* in a variety of cancers, including prostate and ovarian cancers (Rimkus et al., 2016; Carpenter and Ray, 2019). Interestingly, Balanophora polyandra Griff (BPPs) plant extract polysaccharides also suppress the Hh pathway through EMT inhibition downstream, *in vivo* and *in vitro*, but the specific mechanism remains largely unknown (Li et al., 2021). Combined SMO and Gli-targeted therapies are currently undergoing preclinical testing (Rimkus et al., 2016), as finding the balance between upstream and downstream regulation could be the key to Hh targeted therapeutics.

Under the third category, Shh ligand is the most abundant of the hedgehog ligands, and therefore is a promising therapeutic target (Rimkus et al., 2016). Shh monoclonal antibody 5E1 has been implicated to inhibit medulloblastoma (Coon et al., 2010) and to reduce pancreatic tumor size (Chang et al., 2013) *in vivo*, but 5E1 has not yet reached human trials (Carpenter and Ray, 2019). RU-SKI 43 inhibits the downstream enzyme Hhat and reduces proliferation of breast cancer and pancreatic cancer cells, as shown in preclinical trials (Carpenter and Ray, 2019). Through both upstream and downstream inhibitors, Hh targeted therapeutics serve as effective strategies to decrease tumor growth and metastasis.

**FIGURE 5:**
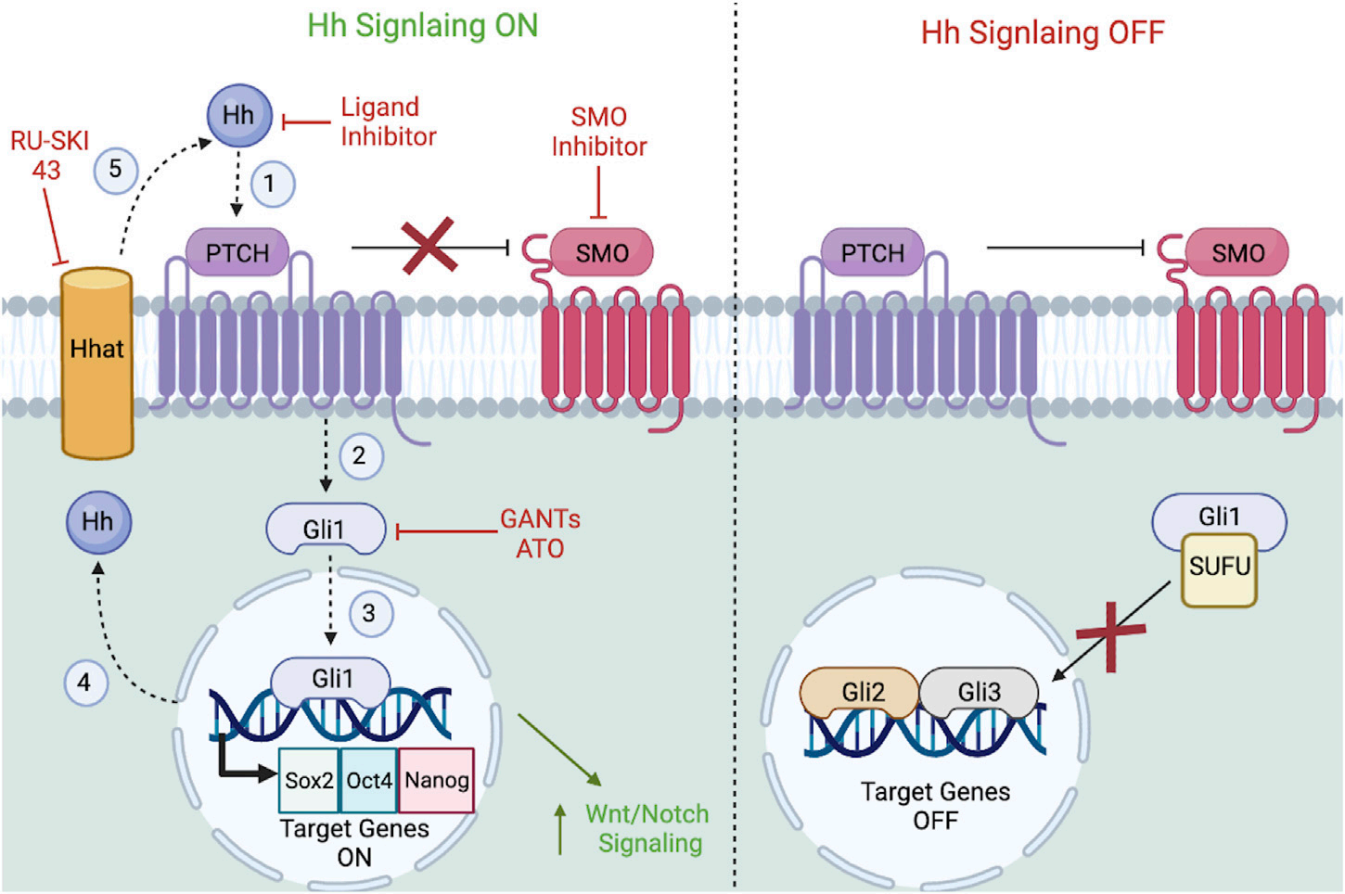
Hh signaling pathway in CSCs. Referring to the left side of the figure, Hh signaling is considered ON when (1) upon Hh ligands binding to the PTCH receptor, (2) signal transduction activates downstream GLI transcription factors, (3) which will translocate to the nucleus to transcribe target genes. (4) After the translation of the Hh protein in the nucleus, membrane-bound Hhat catalyzes palmitoylation of Hh ligands, (5) so that it can bind to PTCH1 in a cyclic fashion. Referring to the right side of the figure, PTCH acts a negative regulator of SMO in the absence of ligand-receptor activation. SUFU will also bind to GLIs and anchor them to the cytoplasm to prevent target gene activation. Featured inhibitors of the Hh signaling pathway include SMO inhibitors, GLI inhibitors, and ligand/enzyme inhibitors. Downstream, Hh ligand-receptor engagement interacts with the Wnt and Notch pathways. Figure constructed via Biorender.com.

The Hh pathway is necessary for the maintenance of CSCs in multiple cancer subtypes. Current research targeting the Hh pathway is focused on SMO inhibitors, GANTs, and ligand/enzyme inhibitors, of which select inhibitors are FDA approved to treat BCC while others are still undergoing pre-clinical and clinical trials. For example, GDC-0449 (Vismodegib), a SMO antagonist that suppresses the activation of downstream Hh target genes, displays anti-tumor activity in locally advanced and metastatic BCC [NCT00607724]. Moreover, Vismodegib, displayed good activity when treating BCC over long-term regimens (>3 months) (Ally et al., 2014; Dréno et al., 2017). However, a phase II clinical trial administering Vismodegib to treat BCC resulted in poor efficacy and multiple adverse events among patients, including muscle spasms (76%), alopecia (58%), and dysgeusia (50%) (Sofen et al., 2015). While there are several unresolved cases regarding the specific role of the Hh signaling pathway, it is known that Hh signaling plays a critical role in cancer progression, and a better understanding of Hh interactions in each tumor sub-type is vital. It is also important to highlight that the Hh pathway may be activated in response to chemotherapy (Merchant and Matsui, 2010) and can overcome chemotherapy resistance in medulloblastomas (Bar et al., 2007) and pancreatic cancer (Olive et al., 2009). Therefore, Hh inhibitors may be better served to combat early-stage tumors or should be administered following surgical removal of the tumor mass. Overall, Hh inhibitor administration during end-stage tumor progression typically correlates with poorer prognosis. In association with this claim, one established paradox in regard to CSC treatment states that positive patient response rates do necessarily correlate to patient survival rates, and the key to novel CSC treatments should be to develop new statistical methods to better asses changes in CSCs at multiple timepoints (Huff et al., 2006). Despite the vast array of clinical knowledge surrounding Hh signaling pathway (Figure 5), a greater understanding of Hh pathway’s role in CSCs is essential for designing combination therapy that utilizes selective Hh-inhibitors, traditional chemotherapeutics, and/or other major CSC signaling pathway inhibitors.

### 3.5 VEGF signaling pathway in CSCs

#### 3.5.1 VEGF ligand-receptor engagement

VEGF is not only necessary for angiogenesis and tumor progression but also acts independently of its angiogenic role to maintain CSCs via paracrine and autocrine signaling (Mercurio, 2019a; Müller et al., 2020). Inhibitors of VEGF may regulate CSCs by decreasing VEGF and subsequently suppressing tumor progression (Müller et al., 2020). VEGF ligands A-E and placental growth factor (PIGF) ligand each activate varying vascular endothelial growth factor receptors (VEGFRs), which each possess seven immunoglobulin-like extracellular domains and an intracellular tyrosine kinase domain (Shibuya, 2011; Takahashi, 2011). There are 3 types of VGFRs: VEGFR-1 (Flt-1), VEGFR-2 (Flk-1/KDR), and VEGFR-3 (Flt-4) (Shibuya, 2011). For instance, VEGF-A regulates angiogenesis through activating VEGFR-1/2, while ligands VEGF-C/D activate VEGFR-3 to initiate lymphangiogenesis, the formation of new lymphatic vessels from pre-existing ones (Shibuya, 2011). Therefore, VEGF-A and VEGFR-1/2 are the classical targets for anti-angiogenic therapeutics (Takahashi, 2011). Upon ligand-receptor engagement, the cell surface receptors will become activated and dimerized through transphosphorylation, resulting in a downstream signaling cascade (Holmes et al., 2007).

#### 3.5.2 VEGF upstream and downstream mechanisms

VEGF not only promotes CSC stemness by stimulating angiogenesis through paracrine signaling but also creates a microenvironment for CSCs through neuropilin1 (NRP1) via an autocrine signaling pathway (Beck et al., 2011). VEGF ligands can also act independently of the VEGFRs via neuropilins1/2 (NRPs), transmembrane glycoprotein receptors largely expressed on tumor cells, which function in tumor initiation and maintenance (Mercurio, 2019a). VEGF-NRP2 triggers the extracellular matrix (ECM) glycoprotein laminin’s engagement with α6β1 integrins on the cell membrane (Goel et al., 2012). Laminin-integrin engagement activates the FAK/Ras pathway, stimulates Hh target gene Gli1, and ultimately enhances the expression of a stem cell factor, BMI-1 (Mercurio, 2019a). The VEGF pathway also activates the Rac1GTPase, which in turn inhibits the Hippo kinase LATS, thus ultimately promoting the activation of TAZ, which can reprogram cancer cells into cancer stem cells (Piccolo et al., 2014; Elaimy et al., 2018). While the classically activated VEGF-VEGFR pathway contributes to angiogenesis and lymphangiogenesis to support the maintenance of the tumor, the VEGF-NRP loop facilitates the self-renewal and persistence of CSCs (Müller et al., 2020; Yang et al., 2020). Most notably, the VEGF-NRP loop results in a positive feedback loop with the Hh pathway, since Gli1 upregulates NRP2 expression (Mercurio, 2019a). Similarly, the crosstalk between the VEGF-NRP loop and Hh pathway is also crucial to Hh signaling that engages the PIGF ligand in the onset and progression of medulloblastomas (Snuderl et al., 2013).

#### 3.5.3 VEGF-associated genes, markers, and molecules

The following genes, markers, and molecules enhance the stemness properties of CSCs via VEGF signaling: 1) C-Myc and Sox2 genes show a strong correlation with VEGFR-2 through activation of JAK2/STAT3 signaling in breast CSCs *in vivo* (Zhao et al., 2015); 2) Hypoxia-inducible factor-1α α (HIF-1α) is a known target gene of VEGF that interacts via the PI3K/Akt pathway (Choi et al., 2011); 3) CD133, a transmembrane protein biomarker found on CSCs, promotes tumor stemness and reoccurrence of HCC through a mechanism dependent on VEGFR2 and Nanog (Liu et al., 2017); 4) COUP transcription factor II (COUP-TFII) is a unique protein that stimulates lymphangiogenesis and tumorigenesis in prostate cancer, enhancing NRP-2 expression (Lin et al., 2010; Qin et al., 2013); and 5) Heparin and NRP1 are involved in VEGFR activation via VEGF-A mechanisms (Takahashi, 2011). Interestingly, some Notch ligands (ex. DLL4) display unique properties in that they may suppress VEGFR2 and NRP1 expression, while other Notch ligands, such as DLL1, stimulate VEGF signaling (Mercurio, 2019a). The following molecules are known to naturally inhibit VEGF signaling, thereby impeding CSCs metastasis: 1) Vitamin C inhibits VEGF signaling through degrading HIF-1α (Zhao et al., 2020); and 2) Melatonin (MLT), a natural pineal gland hormone, regulates neoangiogenesis and inhibits lung and breast cancer cells through the downregulation of HIF-1α/ROS/VEGF (Cheng et al., 2019). While VEGF activates the Raf/MEK/ERK and PI3K/Akt pathways downstream (Ferrara et al., 2003), additional studies are warranted to explore the distinct crosstalk between VEGF and its associated genes and molecules, such as Notch and JAK-STAT signaling components, to unravel novel CSC-targeted therapeutics.

#### 3.5.4 VEGF signaling and cancer: preclinical and clinical studies

The following VEGF targeted therapeutics are promising therapeutics for inhibiting CSCs. Targeted VEGF pathway therapies fall into one of four categories: 1) VEGF ligand inhibitors, 2) receptor inhibitors, 3) VEGF decoy receptors, and 4) ribozymes targeting VEGF (Cardones and Banez, 2006).

Under the first category, bevacizumab (Avastin) is an anti-VEGFA ligand inhibitor approved by the FDA to treat metastatic CRC and renal cell carcinomas (RCC) in combination with traditional therapeutics (Cardones and Banez, 2006). Bevacizumab is currently undergoing phase II clinical trials to treat advanced stage prostate cancer in combination with traditional chemotherapeutic Docetaxel [NCT00574769]. Despite bevacizumab being the most common anti-VEGF drug by blocking VEGFR tyrosine kinases it also enhances VEGF/NRP signaling, thereby resulting in a “VEGF paradox”: bevacizumab cannot fully eradicate prostate CSCs since the drug will enrich CSCs while primarily targeting non-CSCs (Ferrara, 2005; Mercurio, 2019a). To this end, VEGFR therapy combined with NRP2 inhibitors is more effective than the stand-alone drug to treat prostate cancer (Mercurio, 2019a). Similar to bevacizumab, microRNA-140-5p is also an anti-VEGFA ligand inhibitor that hinders breast CSC tumor metastasis and angiogenesis both *in vitro* and *in vivo* (Lu et al., 2017). The most common anti-VEGF strategies have focused on molecules and ligands aimed at diminishing VEGFR action, but the downside to this approach is that VEGFRs bind to multiple ligands within their super-family (Ferrara, 2005). Ligand targeted therapeutics should not only diminish non-CSC tumor cells but also eradicate CSCs by hindering the VEGF-NRP loop.

Under the second category, VEGFR tyrosine kinase (TRK) inhibitors hinder angiogenesis and tumor growth (Takahashi, 2011). Sunitinib, a VEGFR tyrosine kinase inhibitor, is FDA approved in an oral tablet form to treat RCCs and gastrointestinal tumors (Takahashi, 2011). Sorafenib is also a tyrosine kinase VEFGR2/3 inhibitor and is FDA approved to treat advanced renal carcinoma and HCC (Takahashi, 2011). Similarly, ramucirumab (Cyramza) is a monoclonal antibody blocks VEGFR2 and is FDA approved to treat multiple solid cancers (Itatani et al., 2018). Vatalanib blocks kinase VEGFR activity and has completed phase II clinical trials with promising results to treat progressive meningioma [NCT00348790] and generalized advanced stage cancers [NCT00171587]. Furthermore, an *in vivo* study of CSC skin papillomas highlighted how anti-VEGFR2 antibodies in combination with NRP1 deletion prevented tumor metastasis and decreased tumor size through impairing CSC stemness properties (Beck et al., 2011). VEGFR inhibitors show promising effects in both clinical and non-clinical studies.

Under the third category of VEGF decoy receptors, Aflibercept (Zaltrap®) is a soluble recombinant fusion protein that acts as a decoy receptor by binding to VEGF-A ligand with high affinity, preventing VEGFR1/2 activation (Ciombor and Berlin, 2014). Aflibercept has been clinically approved to treat metastatic colorectal cancer in combination with traditional therapeutics (Ciombor and Berlin, 2014), and is currently undergoing phase II clinical trials to treat esophageal and gastric cancers [NCT01747551]. Although effective in the short-term, anti-VEGF therapy is not stable, thus new approaches involving VEGF splicing (Lee et al., 2018) and co-adjunctive treatment with anti-vascular mimics have been explored (Liang et al., 2021b).

Under the fourth category, Angiozyme, a synthetic ribozyme-based VEGFR1 inhibitor cleaves site-specific RNA molecules, which downregulates Flt-1 and KDR, paving the way for a new class of VEGF therapeutics (Weng and Usman, 2001; Weng et al., 2005). A phase I dose escalation study of Angiozyme on patients with refractory tumors showed promising results (Weng et al., 2005), and a phase II clinical trial on metastatic kidney cancer patients was also well tolerated [NCT00021021]. In *in vitro* studies, anti-VEGF_165_ ribozyme showed 90.7% efficacy in anti-angiogenesis gene therapy for the treatment of tumors (Gu et al., 2004). More research should be conducted to better understand the promising effects of this novel class of anti-VEGF ribozyme therapeutics.

**FIGURE 6:**
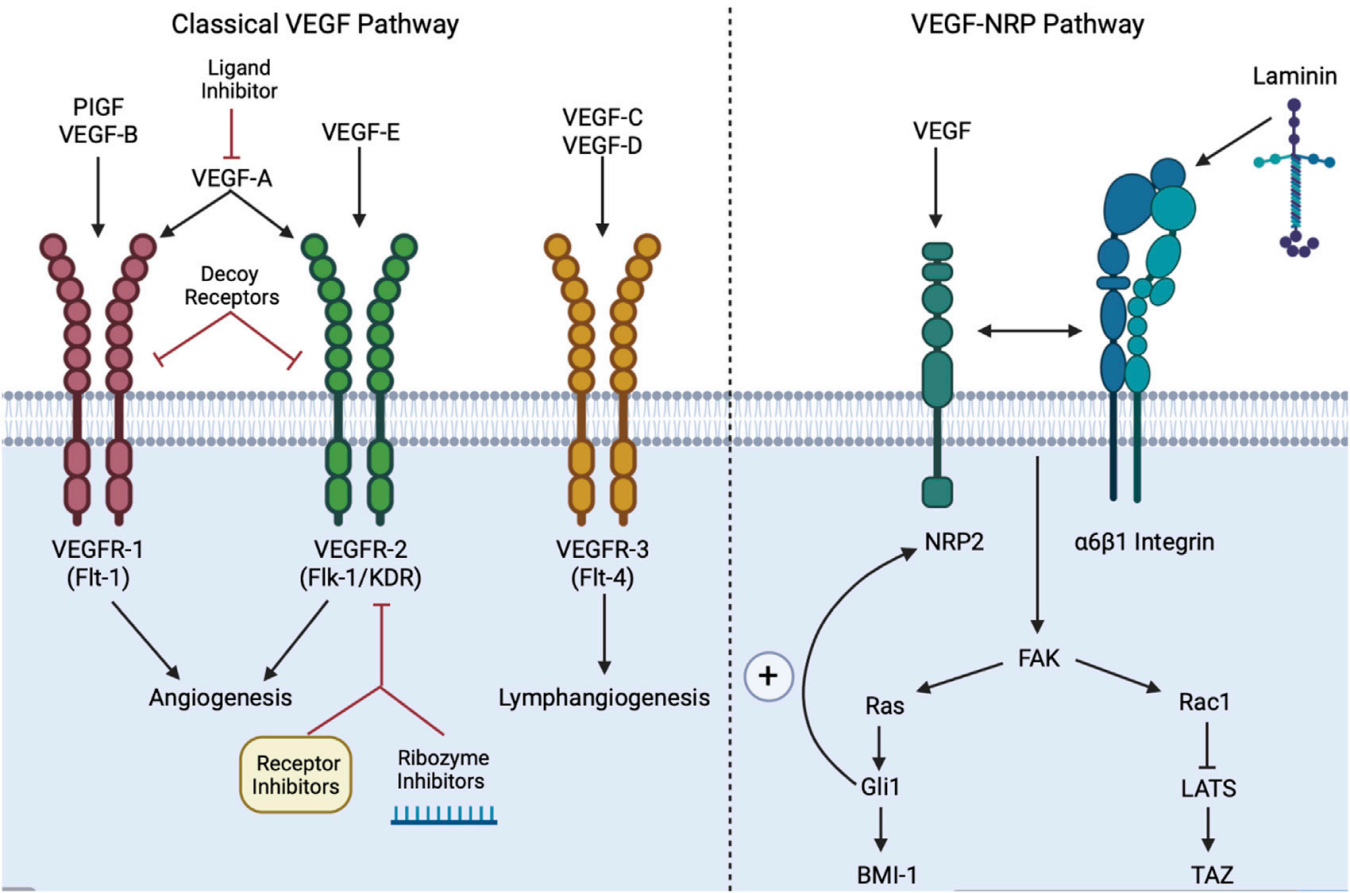
VEGF signaling pathway in CSCs. Referring to the left side of the figure, VEGF ligands A-E and PIGF ligand each activate varying VEGFRs, i.e., VEGFRs 1-3. Upon ligand-receptor engagement, tyrosing kinase receptors dimerize and transphosphorylate resulting in VEGFR-1/2 activating angiogenesis and VEGFR-3 initiating lymphangiogenesis. Referring to the right side of the figure, VEGF ligands can also act in an autocrine fashion via NRP1/2. Specifically, NRP2 triggers laminin engagement with α6β1 integrins, triggering the FAK/Ras pathway, which branches off into two downstream mechanisms: (1) FAK protein tyrosine kinase activates Ras, followed by Hh target gene Gli1, and ultimately enhances the expression of a stem cell factor, BMI-1; or (2) FAK activates Rac1 GTPase, which in turn inhibits the Hippo kinase LATS, thus ultimately promoting the activation of TAZ, permitting the turnover of non-cancer cells into cancer cells. Featured inhibitors of the VEGF signaling pathway include ligand inhibitors, receptor inhibitors, decoy receptors, and ribozyme inhibitors. Figure constructed via Biorender.com.

Both the classical VEGF pathway and VEGF-NRP pathway promote the progression of CSCs within multiple cancer subtypes. Targeting VEGF signaling in combination with chemotherapeutic drugs that target non-CSC pathways can potentially overcome therapy resistance. While VEGF pathway inhibitors are typically well tolerated via multiple routes of administration, common toxicities include fatigue, hypertension, proteinuria, hypothyroidism, and deficient wound healing (Kieran et al., 2012). Further studies are ongoing to address the more serious adverse events that can occur as a result of anti-VEGF related therapies [NCT05108519, NCT04788381], namely thrombosis, encephalopathy, deficient growth plate formation, wound healing deficiencies, and death (Kieran et al., 2012). Current research targeting the VEGF pathway is focused on ligand inhibitors, receptor inhibitors, VEGF decoy receptors, and ribozymes targeting VEGF, of which select inhibitors are marketed to treat CRC, RCC, HCC, solid tumors, and gastrointestinal tumors. Clinical trials also highlight how VEGF combination therapy (i.e., anti-VEGF drug in combination with traditional chemotherapy) is more effective than standard therapy. For example, the most studied FDA approved anti-VEGF inhibitor, bevacizumab, is currently in phase III trials in combination with capecitabine to treat metastatic breast cancer and showed increased response rate from 9.1% to 9.8% (*p* = 0.001) (Miller et al., 2005). Similarly, another phase III study on bevacizumab and paclitaxel for the treatment of breast cancer provided further supporting evidence of improved objective response rates (Ho and Kuo, 2007). However, positive response rates to combination therapy may not necessarily transfer to overall survival rates of the patient. Moreover, although bevacizumab is widely used to treat CRC and RCC, as of 2010 bevacizumab’s breast cancer indication has been withdrawn (Sasich and Sukkari, 2012). The FDA withdrawing bevacizumab’s indication highlights the major hurdle of anti-angiogenic therapies at treating an array of heterogenic cancer subtypes. As stated above, perhaps the reason for bevacizumab’s inefficacy at treating breast cancer is the VEGF paradox; the paradox states that anti-VEGF drugs commonly target the classical pathway while simultaneously upregulating the VEGF-NRP pathway, which supports CSC maintenance. In accordance with this paradox, it may be more beneficial to treat early tumors with anti-angiogenic drugs that inhibit tumor vascularization, rather than to treat highly vascularized, late-stage tumors, but research on early-stage anti-VEGF treatments is currently limited. Clinical trials on the VEGF signaling pathway (Figure 6) should be expanded upon to encompass the unique signaling mechanisms involved in CSC function.

### 3.6 TGFβ-Smad signaling pathway in CSCs

#### 3.6.1 TGFβ ligand-receptor engagement

Under normal conditions the TGF-β signaling pathway plays a vital role in cell differentiation, homeostasis, and organism development (Yang et al., 2020). However, when downstream signaling proteins are mutated, TGF-β suppresses the development of immune cells and allows for the metastasis and growth of prostate, lung, and breast cancer CSCs (Bellomo et al., 2016; Futakuchi et al., 2019). Upon ligand binding, a type II TGF-β ligand bound receptor will phosphorylate a type I receptor, and the T*β*RII–T*β*RI receptor complex will ultimately phosphorylate receptor-regulated Smads (R-Smads) to transcribe targeted genes in the nucleus (Yang et al., 2020). There are three categories of Smads: 1) receptor-regulated Smads are either activated by activin, a beta subunit dimer of the TGF-β protein superfamily, termed activin TGF-β (i.e., Smads 2/3) or are activated by BMP (i.e. Smads1/5/8/9), 2) coSmad (i.e. Smad4), is common to multiple TGF-β signaling pathways, and 3) i-Smads (i.e. Smad6/7) will inhibit the signal transduction of the TGF-β family (Yang et al., 2020). While the canonical TGF-β signaling pathway utilizes Smad2/3 to regulate gene transcription, the non-canonical TGF-β signaling pathways involves Smad 1/5 and can lead to the downstream activation of MAPK/ERK pathway proteins and transcription factors, which are involved in fibrosis and tumorigenesis (Clayton et al., 2020; Finnson et al., 2020; Guo et al., 2020). The non-canonical pathway also leads to the activation of the PI3K/Akt pathway, which is one of the most frequently over-activated downstream intracellular pathways that is involved in several human cancers (Rascio et al., 2021). Other molecules activated by the non-canonical TGF-β signaling pathways include NF-κB, Rho/Rac1, Cdc42, focal adhesion kinase (FAK), Src, and Abl (Neuzillet et al., 2013). The T*β*RII–T*β*RI receptor complex will also activate other non-Smad proteins signals, such as ubiquitin ligases, or small GTPases, resulting in a diverse molecular cascade (Bellomo et al., 2016). The TGFβ pathway is a potential avenue for cancer therapeutics due to its vital role in homoeostasis, induction of inflammatory cytokines in the ECM and regulation of T and B lymphocytes (Bellomo et al., 2016). Non-cellular components are mainly defined by the ECM, which make up the bulk of the stem cell niche (Pattabiraman and Weinberg, 2014). In this way, the TGF-β pathway monitors CSCs, and aberrant TGF-β signaling results in improper function of the immune response.

#### 3.6.2 TGFβ upstream and downstream mechanisms

It is important to highlight TGF*β*’s dual function—at times inhibiting and at other times promoting CSCs progression. Interestingly, in the early stages of cancer, TGF-β suppresses tumors, but in the later stages, TGF-β promotes tumor growth and survival (Futakuchi et al., 2019). On one hand, in the bone microenvironment, TGF-β leads to liver, gastric, melanoma, prostate, renal, glioblastoma, leukemia and bladder cancer proliferation via the downstream activation of the MAPK/ERK and PI3K/Akt, and induces breast cancer metastasis via activation of the Wnt pathway (Bellomo et al., 2016; Futakuchi et al., 2019). TGF*β features an* autocrine loop via its downstream targets Sox4, Oct4, and *Sox*2, and disruption of the TGF*β* pathway would hinder the progression of glioma CSCs (Ikushima et al., 2009). TGFβ is also implicated in cancer progression by inducing EMT and attenuating the anti-tumorigenic effects of dendritic cells, natural killer (NK) cells, CD8^+^ T cells, and CD4^+^ T cells, which can be further subdivided into pro-inflammatory TH1 and anti-inflammatory TH2 cells (Akhurst and Hata, 2012; Kim et al., 2021a; Kim et al., 2021b). On the other hand, TGF*β also inhibits* ALDH1 on CSCs, thereby limiting their self-renewal capacity and halting tumor progression (Bellomo et al., 2016). Along these lines, TGFβ may act as a double-edged sword, wielding both pro- and anti-inflammatory effects by driving TH17 via secretion of IL-6 (Akhurst and Hata, 2012).

Moreover, Nodal and Activin are *TGFβ* ligands can both signal through the same receptors and in many cases the effects are indistinguishable from each other in that both Activin and Nodal and share the downstream effectors Smad2/3 (Pauklin and Vallier, 2015). Under normal conditions, Nodal ligand plays a vital role in embryogenesis, namely inducing epiblast implantation and guiding left-right axis neurulation (Pauklin and Vallier, 2015). Consistent with Nodal’s role in embryogenesis, Activin/Nodal signaling maintains pluripotency in stem cells through induction of Nanog and other cell cycle factors (Pauklin and Vallier, 2015). Cripto-1 is similar to Nodal in that it is an embryonic protein involved in TGF-β signaling (Arboretto et al., 2021). Both Cripto-1 and Nodal have minimal expression in terminally differentiated cells, but are often re-expressed in CSCs and in a dose-dependent manner can trigger more aggressive phenotypes and worsened prognosis for several cancer-subtypes (Arboretto et al., 2021).

Due to the complexity of the TGF*β’s* highly context-dependent downstream mechanisms, developing therapeutics should first identify the stage of cancer progression optimal for a personalized, patient-specific treatment plan, which balances TGFβ’s pro-inflammatory and anti-inflammatory effects.

#### 3.6.3 TGFβ associated genes, markers, and molecules

The following genes and markers are known to upregulate the stemness properties of CSCs via TGF-β/Smad signaling: 1) TGF-β1 in low concentration over short time periods upregulate breast CSCs (Hariyanto et al., 2021), 2) cancer upregulated gene (CUG) 2 promoters associated with YAP1 increase EMT in lung CSCs (Kaowinn et al., 2019), 3) cyclin D1 activation of Smad2/3/4 increases liver CSCs self-renewal and stemness (Xia et al., 2017), 4) CD51 increased migratory and invasive potential in colorectal CSCs (Wang et al., 2017), 5) CD133 acts via a Smad-dependent transcriptional mechanism in HCC, melanoma (Korn et al., 2021), and ovarian CSCs (Ikram et al., 2021), and 6) CD44/CD44v integrates with TME signals to upregulate HCC CSCs (Yan et al., 2015; Bellomo et al., 2016; Yang et al., 2020). Targeting these known upstream molecules, will result in inhibition of the TGF-β/Smad signaling pathway and could serve as potential therapeutics. Within the bone microenvironment, TGF-β is also known to induce the parathyroid thyroid hormone-related peptide (PTHrP) downstream, which stimulates osteoclast activity to destroy bone and promote tumor growth and metastasis (Futakuchi et al., 2019). Currently, the following molecules are known to inhibit TGF-β/Smad signaling: 1) miR-106 targeting Smad7 inhibits gastric CSCs and is implicated as a potential biomarker in CRC, and 2) Dkk-3 inhibits matrix metallopeptidases 9/13, which are downstream TGF-β-induced enzymes, implicated in preventing prostate CSC metastasis (Peng et al., 2020; Yang et al., 2020). Further investigation will uncover the distinct crosstalk between TGF-β and the TME, leading to breakthrough immunotherapeutics.

#### 3.6.4 TGFβ signaling and cancer: preclinical and clinical studies

TGF-β is a valuable target in oncology that should be regulated accordingly. Current drugs on the market that inhibit TGFβ signaling include angiotensin type II receptor inhibitors (i.e. losartan and candesartan) (Akhurst and Hata, 2012). Drugs targeting the TGF-β pathway under clinical trials include anti-ligand antisense oligonucleotides (ASOs), competitive ligand/receptors, antibodies, kinase inhibitors, and small molecule inhibitors (SMIs) (Akhurst and Hata, 2012).

TGFβ antibody therapy has proven to be very effective; since TGFβ can induce an inflammatory TME thereby lowering the efficacy of cancer and radiotherapy (RT) treatments, inhibiting the TGFβ pathway is likely to directly enhance the efficacy of RT (Chen et al., 2021a). Indeed, TGFβ receptor 2 (TGFβR2)-neutralizing antibody MT1 and the small molecule TGFβR1 inhibitor LY3200882 in combination with RT results in increased anti-tumor efficacy against murine orthotopic models in HNSCC (Hamon et al., 2022). The TME is composed of an array of different immune cells and proteins that support the development of cancer cells, including CSCs (Arneth, 2019). The mechanism through which TGFβ interacts with the TME is closely connected to CSCs, in part providing the inflammatory microenvironment, which plays a critical role in tumorigenesis, tumor progression, and metastasis (Zhang et al., 2018b)**.**

In preclinical studies, TGF-β can be directly inhibited. For example, through receptor kinase inhibitors, TGF-β signaling plays a vital role in the bone microenvironment, which harbors significantly more SOX2, CD44, and CD166 positive CSCs than those in the subcutaneous (subQ) microenvironment (Futakuchi et al., 2019). Treatment with TGF-β R1 kinase inhibitor (R1-Ki) in mouse mammary tumor cells reduced tumor volume in the bone microenvironment via TGF-β activation of ERK 1/2 and Akt pathways, but BMP signaling did not contribute to tumor growth (Futakuchi et al., 2019). Similarly, another R1-Ki, SD-208, suppressed the development of melanoma bone metastasis by blocking TGF-β induction of Smad3 phosphorylation (Mohammed et al., 2016). Other studies, which not only block the TGF-β Nodal/Activin Alk4/7 receptor but also simultaneously inhibit the Hh pathway, abolish CSC’s self-renewal capacity and render CSCs more susceptible to gemcitabine *in vivo* (Lonardo et al., 2011). Monoclonal antibodies that interfere with TGF-β proteins Cripto-1 or Nodal *in vitro* have also shown promising effects at abrogating CSCs (Arboretto et al., 2021). Moreover, SB431542, a SMI, partially reversed Nodal-induced chemoresistance in melanoma CSCs, *in vitro* (Li et al., 2018b). Therefore, the majority of TGF-*β* cancer therapeutics work to downregulate rather than upregulate TGF-β.

Rather than inhibiting TGF-β, treatment with TGF-*β* resulted in a significant decrease in proliferative CSC cytokines (i.e., cytokeratin-14, frizzled-7) and saw an increase in markers for slow proliferation (i.e., mucin-1 and cytokeratin-18) (Bellomo et al., 2016). Similarly*, in vivo* TGF*β*’s activation of Smad2/3 inhibited ABCG2, a chemotherapy efflux transporter, leading to reduction of gastric CSCs and overall reduction in tumor size (Ehata et al., 2011). Therefore, due to the dual role of TGF-β in the immune response, at times benefiting and at times hampering the growth of CSCs, we must turn to personalized medicine.

In the clinic, ample consideration of the patient’s genetics and specific biomarkers may guide the development of effective TGF-β treatments. While many TGF-β treatments aim to discover patient-specific anti-TGF-β therapy, there is growing interest in assessing patients based on their unique TGF-β tumor response and relevant oncogenic pathways in cancer cells (Chen et al., 2021c). Such strategy requires probing germline genetic variation between individuals, which should further guide the identification of patients likely to respond to anti-TGF-β therapy (Chen et al., 2021c). Besides looking into patient genetics, TGF-β biomarkers include circulating levels of TGFβ, levels of P-SMAD2 levels in the peripheral blood mononuclear cells (PMNCs), and USP15 in glioblastoma breast and ovarian cancer (Akhurst and Hata, 2012; Eichhorn et al., 2012). Biomarkers serve as a non-invasive way of providing targeted therapeutic treatment. Moving forward, the goal of TGFβ drug development should be to enhance the tumor-suppressing effects of TGFβ while inhibiting TGFβ’s role in CSC progression.

**FIGURE 7:**
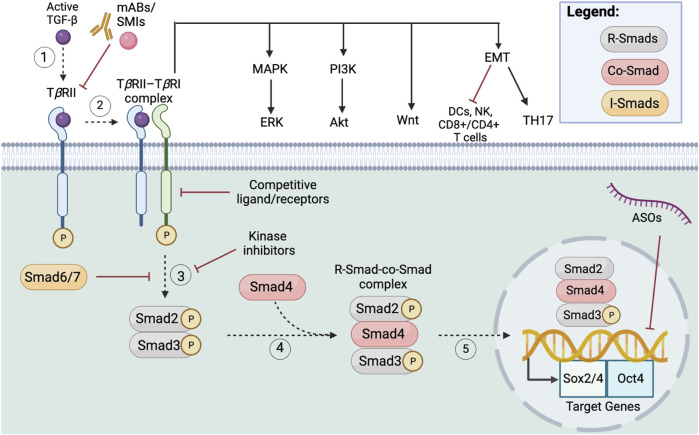
TGF-β-Smad signaling pathway in CSCs. (1) Upon the active TGF-β ligand binding to a type II receptor, (2) there is phosphorylation of the type I receptor, and (3) the T*β*RII–T*β*RI receptor complex will ultimately phosphorylate R-Smads. (4) The R-Smads will form a complex with co-Smad 4 and (5) translocate to the nucleus to transcribe the known targeted genes (Sox2/4, Oct4). Featured inhibitors of the TGF-β pathway are SMI mAbs, ligand-receptor inhibitors, kinase inhibitors, upregulation of the endogenous i-Smad6/7, and ASOs. Downstream of ligand-receptor engagement, TGF-β will upregulate the MAPK/ERK, PI3K/Akt, and Wnt pathways. In terms of the TME, TGF-β upregulates TH17 secretion while downregulating dendritic cells, NK cells, CD8^+^/CD4^+^ T cells. Figure constructed via Biorender.com.

The development of cancer therapeutics that target the TGF-β pathway have demonstrated satisfactory safety and efficacy in cancer patients. However, TGF-β’s dual role in cancer progression should be further explored to develop superior patient-specific anti-TGF-β treatments. Moreover, since TGF-β is ubiquitously expressed in multiple cell types to regulate cell homeostasis and organism development, there is an inherent physiological barrier to treatment. Current anti-TGF-β treatments including ASOs, competitive ligand/receptors, antibodies, kinase inhibitors, and SMIs are limited to pre-clinical and phase I-II clinical trials. Due to the limited dual role of TGF-β functioning as both a tumor promoter and tumor suppressor, the development of safe and effective TGF-β antagonists remains a challenge. For example, in a phase II study the SMI, galunisertib, in combination with the chemotherapeutic lomustine, failed to efficiently treat glioma compared to the placebo [NCT01582269], and galunisertib must be closely monitored to avoid cardiac toxicity (Kovacs et al., 2015). Furthermore, selective TGF-β biomarkers are only in the initial stages of clinical development. While TGF-β treatment alone is limited, combination therapy has proven to be more effective at targeting not only non-CSCs but also ablating CSCs. For example, Vactosertib, a TGF-β kinase inhibitor, in combination with chemotherapy has proven to be well tolerated in phase I-II clinical trials to treat solid tumors [NCT02160106], metastatic colorectal or gastric cancers [NCT03724851, NCT03698825], non-small cell lung cancer [NCT03732274], and urothelial cancer [NCT04064190]. In conclusion, drugs that target the TGF-β pathway are highly valuable to the cancer research field, as they boast improved patient response when compared to classical cancer treatments. While there are still many hurdles that TGF-β therapies must overcome before reaching the market, expanding knowledge of targeted CSC treatments are enhanced by continued TGF-β research (Figure 7)*.*

### 3.7 Accessory CSC signaling therapeutics

CSCs have also been targeted by inhibition of IL-8 treatment, which prevents the recruitment of MSCs (Müller et al., 2020). IL-8 production is closely associated with the expansion of CSCs in the TME (Kim and Kahn, 2014) and its activation has been implicated in the proliferation of CSCs in the highly aggressive triple-negative breast cancer (Hirata et al., 2022), suggesting the use of IL-8 inhibitors to sequester CSCs. CSCs can also be regulated through blockade of GM-CSF, which suppresses the recruitment of TAMs (Müller et al., 2020). There are two main lineages of macrophages that are known to interact with CSCs. M1, which are classically activated, pro-inflammatory macrophages that induce and maintain CSCs through the production of IL-6. Conversely, M2 are alternatively activated, anti-inflammatory macrophages (Tsuchiya and Shiota, 2021). In leukemic mice, TAMs are shown to significantly contribute to the TME with bone marrow-derived macrophages becoming polarized into leukemic cells following injection of leukemic cells into the animals, indicating that targeting TAMs may retard the onset and progression of tumors (Li et al., 2020). Of note, the use of GM-CSF and TAM (tumor associated macrophages) inhibitors, like CSF-1R, have shown promising results in blocking the crosstalk between macrophages and tumor cells (Liu et al., 2016; Li et al., 2020).

Conversely, strategies designed to indirectly target CSCs by modulating non-CSCs have also demonstrated some success in reducing tumor growth. For example, BMP and Gremlin decrease TGFβ and target non-CSC state polarization (Pattabiraman and Weinberg, 2014). The BMP-antagonist, Gremlin 1 or GREM1, is closely linked with metastasis, specifically stemness of breast cancer cells (Ren et al., 2019), and accompanies the poor prognosis of patients with estrogen receptor-negative breast cancer (Neckmann et al., 2019), suggesting that Gremlin stands as a potent therapy for abrogating cancer cell (i.e., CSC) proliferation. Another approach for indirectly arresting CSCs is via specific CTLA-4 inhibitor, ipilimumab and anti-PD-1, nivolumab, both of which increase T cell cytotoxicity and shows success in treating melanoma and leukemia (Pattabiraman and Weinberg, 2014; Greiner et al., 2020). Targeting leukemic progenitor and stem cells by specific cytotoxic T lymphocytes can dampen leukemia-associated antigens that mediate immune responses against colony-forming cells including leukemic progenitor cells, which are thought to correspond to the source population of relapse of the disease (Greiner et al., 2020). Altogether, promising results in the laboratory and early limited clinical trials that target the proteins and signaling pathways implicated in CSC proliferation appear to sequester the aberrant self-renewal of CSCs with corresponding reduction in tumor size and prevention of relapse.

### 3.8 Crosstalk of CSC signaling pathways and combination therapy

The signaling pathways Wnt, TGFβ, Notch, JAK-STAT, Hh, and VEGF often interact with each other to maintain and regulate CSCs. Hh and VEGF targeted therapeutics have made great strides in the field of CSC-directed therapeutics as they boast multiple FDA approved drugs, namely Odomzo®, Avastin®, Cyramza®, and Zaltrap® [NCT04066504] (Cardones and Banez, 2006). Conversely, Wnt targeted therapeutics are typically halted at phase I/II due to the complexity of the Wnt pathway (Katoh, 2017). Additionally, it is important to synthesize the connection among the major CSC signaling pathways, as the best mode of treatment may be to target multiple signaling pathways.

The canonical Wnt pathway is known to display crosstalk with the Hh pathway and *vice versa* (Katoh, 2017; Yang et al., 2020). For example, β-catenin upregulates Hh signaling via stabilization of Gli mRNAs, but β-catenin can also lead to the proteasomal degradation of Gli (Noubissi et al., 2009; Zinke et al., 2015). In turn, Hh signaling can also suppress Wnt signaling via induction of Gli 1/2 mediated Hh target gene, soluble frizzled-related protein 1 (sFrP1) (He et al., 2006). Conversely, Gli1 also activates Wnt2b, Wnt4, Wnt7b genes, which upregulate Wnt signaling (Li et al., 2007). This leads to the question as to why Hh CSC targeted therapeutics are more effective than Wnt targeted therapeutics, given the intimate crosstalk between Wnt and Hh signaling. Perhaps, the interplay between Wnt and Hh signaling should also be viewed in accordance with Notch signaling, a key mediator between Wnt and Hh signaling (Koury et al., 2017). Specifically, in colorectal cancer cells, Notch’s Jagged 1 ligand elevates β-catenin activity to drive Wnt signaling (Pannequin et al., 2009). Wnt signaling also directly upregulates Notch signaling components, namely DLL1, Hes1, Notch 2, and Jagged 1 (Kumar et al., 2021). Moreover, Notch’s target gene, Hes 1, regulates Hh signaling in glioblastomas, and Hh signaling can also directly modulate Notch signaling through inducing the same Hes1 gene, as well (Wall et al., 2009; Schreck et al., 2010). Through inhibiting Notch and Hh signaling via combination therapy, prostate cancer CSCs were more sensitive to chemotherapy *in vitro* (Domingo-Domenech et al., 2012). In addition, phase II clinical trials are currently being carried out with Hh pathway inhibitor (Vismodegib) and Notch pathway inhibitor (RO4929097) that show promising results for the treatment of advanced stage sarcoma (Gounder et al., 2022) [NCT00833417]. Future studies should aim to unravel the unique relationship between Wnt, Hh, and Notch in order to develop novel CSC combination therapeutics.

Wnt signaling pathway is also displays crosstalk with TGF-β through a variety of mechanisms. TGF-β and Wnt signaling pathway transcription factors, namely Smad and Lef, respectively, synergistically regulate a set of shared target genes (Guo and Wang, 2009). TGF-β and Wnt also display protein-protein interaction in the cytoplasm, via the binding of Smad7-Axin, and can reciprocally regulate ligand production (Guo and Wang, 2009). The crosstalk between TGF-β and Wnt signaling serve as a potential cancer therapeutic. For example, SMI A83-01, which inhibits TGF-β-induced upregulation of Wnt3, enhances trastuzumab treatment for targeting breast cancer cells *in vitro* (Wu et al., 2017). Current clinical trials that combine Wnt and TGF-β inhibitors are limited.

JAK-STAT pathway displays multiple diverse mechanisms, which overlap with TGF-β and Notch pathways. For instance, in pancreatic ductal carcinoma TGF-β inhibited and IL-1 induced JAK/STAT signaling cascade *in vitro* (Biffi et al., 2019). In comparison, in hematopoietic SCs, TGF-β increased IL-6 mediated STAT3 activation (Tang et al., 2017). Moreover, Notch signaling activates IL-6 induced JAK/STAT signaling in breast cancer tumors (Jin et al., 2013). Current clinical trials featuring combination therapy aim to decipher variations in targeting JAK family members and STATs, rather than targeting multiple signaling pathways. For instance, one clinical trial in phase I for the treatment of lung cancer features Afatinib plus Ruxolitinib combination therapy, which targets IL-6 receptor and JAK1/2 [NCT02145637].

Lastly, VEGF signaling is also vital for Wnt and Notch pathways. VEGF induces Notch signaling, while Notch signaling also modulates the VEGF pathway. Specifically, *in vivo* and *in vitro* studies confirm that VEGF will upregulate Notch pathway modulators, namely DLL1/4, Notch 1, and ADAM-10 (Li and Harris, 2009). Notch signaling also regulates VEGF ligand expression (VEGF and PLGF) and VEGF receptor expression (NRP1/2 and VEGFR1/2/3) (Li and Harris, 2009). Moreover, another study *in vitro* proved that VEGFR-1 kinase activity is required for Wnt/β-catenin CRC cells (Zeitlin et al., 2009). Current VEGF combination therapeutics are in phase IV clinical trials and display promising results for ablating CRC using anti-VEGF drugs plus traditional chemotherapeutics [NCT01972490]. Other VEGF combination therapeutics are in phase II trials and feature anti-VEGF drugs (Bevacizumab) in combination with a DNA synthesis and cell cycle inhibitors (Gemcitabine, Paclitaxel) [NCT00403130]. Future VEGF therapeutics should target not only the VEGF pathway but also inhibit Notch signaling as a potential means for CSC directed therapeutics.

Wnt, TGFβ, Notch, JAK-STAT, Hh, and VEGF signaling are vital for CSC regulation and restoring the pathways to equilibrium is a potential novel avenue for cancer therapeutics. Moreover, a better understanding of these key pathways’ genes, markers, and molecules will allow us to better assess unique therapeutic targets (Table 1). While most clinical trials are focused on targeting one singular pathway in combination with traditional chemotherapy, a more effective approach would be to curate combination products that blocks multiple CSC signaling mechanisms (Table 2). Moreover, while the Wnt pathway is the most complex and centralized among each signaling pathway, targeting signaling pathways that are more downstream of Wnt will result in more specific therapeutics.

**TABLE 1:** CSC signaling pathways’ associated genes, markers, and molecules.

Signaling pathway	Associated factors that enhance CSCs	Associated factors that inhibit CSCs	Cited
Wnt	CTNNB1	AXIN2	Hong (2016)
	APC mutations	APCDD1	Komiya and Habas (2008), Duchartre et al. (2016), Wiese et al. (2017), Ackers and Malgor (2018)
	AXIN1/2 mutations	DKK1	Guan and Fierke (2011), Liu et al. (2013a), Mazzoni and Fearon (2014), Fang et al., 2016; Katoh (2017), Aghabozorgi et al. (2019), Lecarpentier et al. (2019), van Schie and van Amerongen (2020), Liu et al. (2022)
	Cyclin-D1		
	C-Myc		
	LGR5		
	EPCAM		
	CD44/CD44v6		
	CD133		
Notch	DLL	microRNA-34a	Tweedell (2017)
	TNFα	miR-200b-3p	Matsuno et al. (1995), Anders et al. (2006), Guijarro et al. (2007), Sato et al., 2007; Purow (2009), Liu et al. (2013b), Garcia-Heredia et al. (2017), Jimeno et al. (2017), Bach et al. (2018), Renz et al. (2018), Deng et al. (2020), Meisel et al. (2020), Wiese et al. (2020), López-Sánchez et al. (2021), Rodon et al. (2021), Segami et al. (2021), Shan et al. (2021)
	Jagged 2	miR-26a	
	Gli3	PER3	
	Notch1	BMP4*	
	*Hes1-7*		
	*Hey1, Hey2 and HeyL*		
	NRARP		
	*Cyclin D1*		
	*DVL1 gene*		
	*ADAM19*		
	BMP4*		
JAK-STAT	STAT3	STAT 1/2	Tweedell (2017)
	Oct4	IFN I/II	Venkatesh et al. (2018)
	Epo	Mir-218	Chiorean et al. (2015), Zhang et al. (2017a), Zhang et al. (2017b), Fukusumi and Califano (2018), Smith et al. (2019), Chen et al. (2021b)
	RBP4	Ajuba	
	HIF-1α miR-500a-3p	VHL	
	TH17		
Hh	CK2α	miR-361-3p	Tweedell (2017)
	RARα2	miR-326	Raz et al. (2012), Furqan et al. (2013), Schulenburg et al. (2015), Rimkus et al. (2016), Schwartz et al. (2017), Li et al. (2018a), Pietrobono et al. (2019), Sasai et al., 2019; Wei (2019), Jiang et al. (2020), Wang et al. (2020)
	PPM1D	BCL6	
	lncHDAC2	RUNX3	
	VASH2*	Arrb1	
	SPOP*	VASH2*	
		SPOP*	
VEGF	C-Myc	DLL4	Jiang et al. (2014), Miele et al. (2017), D’Amato et al. (2014), Doheny et al. (2020), Gampala et al. (2021), Incardona et al. (1998), Tremblay et al. (2009)
	Sox2	Vitamin C	
	HIF-1α	MLT	
	CD133		
	COUP-TFII		
	Heparin		
	NRP1		
	DLL1		
TGFβ-SMAD	TGF-β1	miR-106	Ferrara et al. (2003), Lin et al. (2010), Choi et al. (2011), Qin et al. (2013), Zhao et al. (2015), Liu et al. (2017), Cheng et al. (2019), Zhao et al. (2020)
	CUG 2	Dkk-3	
	Cyclin D1		
	CD51		
	CD133		
	CD44/CD44v		

This table outlines specific genes, markers, and molecules that either upregulate or downregulate CSCs based on interactions with their respective signaling pathway. Specific regulators denoted as [x]* are known to both upregulate and downregulate CSCs.

**TABLE 2:** CSC signaling pathways and associated therapeutics.

Signaling pathway	Postulated mechanism	Proposed therapeutic drugs and effect		Cited
Wnt	Genetic alterations in the Wnt pathway, specifically the stabilization of β-catenin, the key transducer of canonical Wnt signals. Wnt signaling initiates CSC and tumor cell population growth	Ligand/receptor inhibitors:	Under phase I clinical trials showing anti-CSC effects in prostate and lung cancer	Pattabiraman and Weinberg (2014)
		Cirmtuzumab		Wiese et al. (2017)
		Rosmantuzumab		Komiya and Habas (2008), Liu et al. (2016), Wiese et al. (2017), Ackers and Malgor (2018), Li et al. (2020)
		Vantictumab		Liu et al. (2013b)
				de Sousa and Vermeulen (2016)
		PORCN inhibitors:	Under phase I/II clinical trials targeting the small molecule PORCN that is required for Wnt ligand-receptor activation. Treats metastatic CRC, pancreatic, breast, HNSCC, esophageal, lung, and cervical cancers	[NCT02413853]
		IWP-2		[NCT02278133]
		WNT974 (aka LGK974)		[NCT05156905]
		ETC-159		[NCT01957007]
		Tankyrase inhibitors:	Under preclinical trials for upregulating the destruction complex via AXIN1/2.	
		AZ1366		
		G007-LK		
		β-catenin inhibitors:	In phase I/II clinical trials for blocking CSC motility in AML and advanced pancreatic cancer	[NCT01606579]
		ICG-001 (PRI-724)		[NCT01764477]
		LF3		
		mAB inhibitors:	In both preclinical and phase I clinical trials to target vital Wnt proteins, specifically treats advanced stage solid tumors and lymphomas	[NCT05279300]
		anti-FZD		[NCT01351103]
		anti-ROR1		
		anti-RSPO3		
		anti-LGR5		
Notch	One or more Notch paralogs confer oncogenic activity, with aberrant Notch activation stimulating CSC proliferation and differentiation. Notch also regulates CSC renewal and modulates CSC-mediated tumor formation and recurrence	GSIs:	Includes classes of peptide isosteres, azepines, and sulfonamides. In phase I-II clinical trials for suppressing glioma, breast, ovarian, and adenocarcinoma cancers subtypes	Wang et al. (2009)
		RO4929097		Tweedell (2017)
		PF-03084014		Venkatesh et al. (2018)
		MRK003		Wiese et al. (2017)
		MK-0752		Purow (2009)
				Anders et al. (2006), Meisel et al. (2020), López-Sánchez et al. (2021)
		mABs inhibitors:	Interferes with Notch ligand-receptor binding or prevents the conformational change required for cleavage. Decreased breast, small-cell lung, ovarian, and pancreatic CSCs *in vivo* and effectively decreased solid tumors in phase I clinical trials	Pannuti et al. (2010)
		Tarextumab (OMP-59R5)		[ NCT0113123]
		Enoticumab (ex. REGN421, SAR153192)		[NCT0087155]
				[NCT01277146]
JAK-STAT	JAK-STAT activation stimulates CSCs via EMT. JAK-STAT also facilitates CSC transition resulting in increased tumorigenic and metastatic ability, and chemoresistance	Cytokine inhibitors:	Used in combination with tocilizumab against IL-6. Showed improved survival rates for ovarian cancer *in vivo*. Enhances cancer treatment in HNSCC, pancreatic cancer, and glioblastoma	Chen et al. (2021a)
		Ruxolitinib		Jang et al. (2016), Feifei et al. (2019), Gao et al. (2020), Gharaibeh et al. (2020), Zhang, 2020; Chen et al. (2021b), Hsu et al. (2021)
		JAK inhibitors:	JAK1/2 inhibitors currently under phase I/II clinical trials to treat AML, prostate, colon, rectal, non-small cell lung cancer, and non-Hodgkin’s lymphoma	Kisseleva et al. (2002), Schindler et al. (2007), Wu et al. (2010)
		Pacritinib		[NCT04757259]
		Cerdulatinib		[NCT01839604]
		Momelotinib		[NCT03394144]
		AZD1480		[NCT01994382]
		STAT inhibitors:	Include SMIs, peptide inhibitors, siRNAs, ASOs that interfere with STAT mRNA, and ODNs. Target STAT3, STAT5, as well as STAT dimerization. Phase I clinical trials for treating metastatic CRC, gastric cancer, and HCC	[NCT04021082]
		Napabucasin		
		PY*LKTK		
		AZD915		
		K562		
		U251		
		A172		
Hh	Hh pathway directs cell proliferation, cell fate determination, EMT and the rearrangement of cells. Inappropriate activation in the adult may initiate CSC and cancer growth. Hh overexpression positively correlates with increased CSC stemness markers, which contributes to tumor progression	SMO inhibitors:	Prevent Gli activation downstream, leading to target gene inhibition and CSC suppression. Most inhibitors in this category are FDA approved to treat BCC, while others are under phase I/II clinical trials to treat breast cancer and medulloblastoma	Chen et al. (2021a)
		LDE225 (Odomzo®)		Smith et al. (2019)
		Erismodegib		Furqan et al. (2013), Schulenburg et al. (2015), Sun et al. (2015), Schwartz et al. (2017), Li et al. (2018a), Pietrobono et al. (2019), Sasai et al. (2019)
		Sonidegib		Gampala et al. (2021)
		Vismodegib		[NCT04066504]
		Steroidal alkaloid cyclopamine		[NCT01878617]
		Gli inhibitors:	Inhibits both Gli1/2 downstream effectors to decrease tumor growth. ATO is FDA approved to treat leukemia. Other inhibitors in this category are under preclinical testing for prostate, colon, and ovarian cancers	[NCT00833417]
		Arsenic trioxide (ATO)		[NCT02195973]
		GANT-61		[NCT01487785]
		Balanophora polyandra Griff		
		Ligand/enzyme inhibitors:	Under preclinical trials to inhibit medulloblastoma, pancreatic, and breast cancer	
		5E1 mAB		
		RU-SKI 43		
VEGF	VEGF signaling provides angiogenic support and vascular permeability to cancer cells, but also contributes to tumorigenesis by aiding CSC self-renewal and maintenance	Ligand inhibitors:	Anti-VEGFA ligand inhibitors. Bevacizumab is approved to treat CRC and RCC, and it is in phase II for prostate cancer. 140-5p is in pre-clinical testing to treat breast cancer	Müller et al. (2020)
		Bevacizumab (Avastin®) microRNA-140-5p		Mercurio (2019a)
		Receptor inhibitors:	Some are FDA approved to treat RCC, HCC, solid tumors, and gastrointestinal tumors. Pre-clinical and clinical studies are also ongoing to treat meningioma and skin papillomas	Tsuchiya and Shiota (2021)
		Sunitinib		Lee et al. (2018)
		Sorafenib		Mirzaei et al. (2021), Liang et al. (2021a), Kim et al. (2020), Qu et al. (2021), Jiang et al. (2014), Miele et al. (2017), D’Amato et al. (2014), Doheny et al. (2020), Gampala et al. (2021)
		Ramucirumab (Cyramza®)		Liang et al. (2021b)
		Vatalanib		[NCT00348790]
		Decoy receptors:	VEGFR1/2 inhibitor. Approved to treat CRC and is currently undergoing phase II clinical trials to treat esophageal and gastric cancers	[NCT00171587]
		Aflibercept (Zaltrap®)		[NCT01747551]
		Ribozymes:	VEGFR1 inhibitor. Phase II clinical trials show treatment of metastatic kidney cancer, and *in vitro* studies treat generalized tumors.	
		Angiozyme		
		Anti-VEGF_165_ ribozyme		
TGFβ-SMAD	Activation of TGFβ-SMAD pathway results in CSC renewal and differentiation leading to tumorigenesis; Induces an inflammatory TME thereby lowering the efficacy of cancer treatments	TGFβ inducer:	Decreased gastric CSCs and CSC cytokines, and reduced tumor size *in vivo*	Incardona et al. (1998), Tremblay et al. (2009), Coon et al. (2010), Carpenter and Ray (2019)
		Direct TGFβ		Holmes et al. (2007), Takahashi (2011), Goel et al. (2012), Piccolo et al. (2014), Elaimy et al. (2018)
		Ligand-receptor inhibitor:	In combination with RT results in increased anti-tumor effects in HNSCC *in vivo*	Xia et al. (2017)
		TGFβR2-neutralizing Ab (MT1)		Hamon et al. (2022)
		Kinase inhibitors:	Reduced tumor volume and melanoma bone metastasis via activation of ERK 1/2 and Akt pathways *in vitro*. Vactosertib is in phase I to treat solid tumors and in phase II for the treatment of non-small cell lung cancer	[NCT02937272]
		R1-Ki		[NCT02937272]
		SD-208		[NCT01373164]
		Vactosertib (TEW-7197)		[NCT02160106]
		Small molecule inhibitor:	LY3200882 in combination with RT results in increased anti-tumor effects in HNSCC *in vivo,* and is in phase I/II for targeting solid tumors and advanced metastatic tumors. LY21557299 is in phase II study to treat pancreatic cancer	[NCT03732274]
		LY3200882 (Eli Lilly)		
		LY21557299 (Galunisertib)		
IL-8	IL-8 upregulation aids in CSC formation and acquisition, as well as maintenance of CSC stemness	IL-8 inhibitors:	Inhibits IL-8 production leading to tumor reduction and susceptibility to chemotherapy. IL-8 via its cognate receptors, CXCR1 and CXCR2, may regulate CSC activity. Undergoing phase I clinical trials for breast cancer	Singh et al. (2013), Jin et al. (2018), Choi et al. (2019), Corrò et al., 2019; Jin (2020), Kim et al. (2021a), Hirata et al. (2022)
		17β-estradiol		[NCT03726931]
		Sulconazole		
GM-CSF	GM-CSF-dependent pathway phosphorylates JAK2 and recruits STAT-3 thereby regulating EMT and conveying CSC stemness	GM-CSF inhibitors:	Decreases CSCs, inhibits angiogenesis and vascularization, and reduction in tumor metastasis. In phase I clinical trials for AML	Aliper et al. (2014), Shi et al. (2018), Li et al. (2020), Liu et al. (2020), Müller et al. (2020)
		lCSF-1R		[NCT00988715]
		streptavidin		
		CSCs vaccine		
BMP	As a member of TGFβ superfamily, BMP is thought to act via TGFβ, thereby aiding in the EMT pathway of CSC and tumor formation	BMP-antagonist:	Decreases CSC stemness properties thereby inhibiting tumor development and propagation, metastasis, drug resistance, and relapse. Targets cervical cancer CSCs *in vitro*	Pattabiraman and Weinberg (2014), Sato et al. (2016), Bosukonda and Carlson (2017), Neckmann et al. (2019), Ren et al. (2019)
		Gremlin 1		

This table outlines the signaling pathways implicated in CSCs’ role in tumor onset, progression, and relapse, and the associated pre-clinical and clinical therapeutics.

## 4 Conclusion and perspective

CSCs are a small subclass of cancer cells with self-renewal and metastatic capacity, resulting in resistance to traditional chemotherapeutics and multidrug therapies (Pattabiraman and Weinberg, 2014). Many clinical trials on CSCs have shown promising evidence for cancer therapy. Signaling pathways, including Wnt, TGFβ-SMAD, Notch, JAK-STAT, Hh, and VEGF, are essential regulators of CSCs. Notably, signaling pathway inhibitors include ligand-receptor inhibitors (Cardones and Banez, 2006), decoy receptors (Ciombor and Berlin, 2014), mAbs (Katoh, 2017), siRNA (Hu et al., 2021), and specific molecule (Akhurst and Hata, 2012) and enzyme inhibitors (Gu et al., 2004; Carpenter and Ray, 2019). Inhabiting these signaling pathways may prove beneficial against cancers. Cancer therapies must navigate the intricacies of CSC signaling pathways to eliminate CSC effectively. CSCs may act differently in different cancer sub-types (Yang et al., 2020), resulting in a less than satisfactory classification of CSC therapeutics. Under normal conditions certain signaling pathway may play an intrinsic role in cell homeostasis (Yang et al., 2020), but the same signaling proteins vital to cell development can result in metastasis and growth of CSCs (Bellomo et al., 2016; Futakuchi et al., 2019). CSCs also share signaling pathways with traditional stem cells (Pattabiraman and Weinberg, 2014), limiting therapeutic potential in cancer treatment. While there are multiple novel avenues for cancer therapeutics, the sheer volume of processes and pathways that contribute to CSC stemness can be daunting, and finding one cure-all treatment may be restrictive. Instead, patient-specific therapies that focus on the patient’s cancer profile and genotypic signal processes may prove promising (Katoh, 2017). Future CSC-targeted therapies should aim to create specific inhibitors rather than generalized signaling pathway inhibitors to decrease the number of side effects associated with traditional, indiscriminate cancer therapies. Natural products that target CSCs should also be studied in the future as they may be effective at targeting CSCs without impairing non-cancerous stem cell (Incardona et al., 1998; Cheng et al., 2019; Zhao et al., 2020). Future studies should also delve into the accumulating evidence implicating a crosstalk between CSCs and immune cells within the TME providing key concepts on signaling mechanisms mediating CSC-mediated tumor formation (Müller et al., 2020) as well as possible immunotherapeutics against cancer (Chen et al., 2021a). An integrated understanding of CSC signaling pathways and immune cell crosstalk factors is expected to provide significant improvements to the current knowledge of cancer pathology and treatment.

## Figures and Tables

**Table:** 

**CSCs**	Cancer stem cells
**TGF-β**	Transforming growth factor-β
**JAK-STAT**	Janus kinase-signal transducers and activators of transcription
**Hh**	Hedgehog
**VEGF**	Vascular endothelial growth factor
**TME**	Tumor microenvironment
**AML**	Acute myeloid leukemia
**SCID**	Severe combined immunodeficient
**CD**	Cluster of differentiation
**ABC**	ATP-binding cassette
**HNSCC**	Head and neck squamous cell
**SIRPα**	Signal regulatory protein alpha
**ALDH1**	Aldehyde dehydrogenase 1
**LOX**	Lysyl oxidase
**SC**	Stem cells
**EMT**	Epithelial-to-mesenchymal transition
**MET**	Mesenchymal-to-epithelial transition
**ZEB1**	Zinc finger E‐box‐binding homeobox 1
**SLUG**	Zinc finger protein SNAI2
**MSC**	Mesenchymal stromal cells
**GM-CSF**	Granulocyte macrophage colony-stimulating factor
**BMP**	Bone morphogenic protein
**PDGFR**	Platelet derived growth factor receptor
**PI3K**	Phosphatidylinositol-3-kinase
**Fz**	Frizzled
**GPCRs**	G-protein coupled receptors
**LRP5/6**	Low-density-lipoprotein-related protein5/6
**PCP**	Planar Cell Polarity
**CBP**	CREB-binding protein
**TCF/Lef**	T-cell/lymphoid enhancer
**APC**	Adenomatous Polyposis Coli
**TNKS**	Tankyrase
**PORCN**	Porcupine5-7
**Dsh/Dvl**	Dishevelled
**MCT1**	Monocarboxylate transporter 1
**PDK**	Pyruvate dehydrogenase kinase
**TCF1**	T cell factor
**CTNNB1**	Catenin beta 1 gene
**RSPO**	R-spondin
**EPCAM**	Epithelial cell adhesion molecule
**APCDD1**	APC downregualted-1 gene
**DKK1**	Dickkopf Wnt pathway inhibitor 1
**CLL**	Chronic lymphocytic leukemia
**LIC**	Leukemia initiating cells
**DSL**	Delta/Serrate/Lag2
**NICD**	Notch’s intracellular domain
**CSL**	CBF1/Suppressor of Hairless/Lag-1
**NO**	Nitric oxide
**ROS**	Reactive oxygen species
**MDSCs**	Myeloid derived suppressor cells
**TAMs**	Tumor associated macrophages
**Tregs**	T regulatory cells
**DTX1**	Deltex E3 Ubiquitin Ligase 1
**DLL4**	Delta-like ligand 4
**TNFα**	Tumor necrosis factor-α
**SCC**	Squamous cell carcinoma
***Hes1-7***	Hairy enhancer of split genes
**NRARP**	Notch-regulated ankyrin-repeat protein
**CRC**	Colorectal cancer
**GSIs**	γ-secretase inhibitors
**mABs**	Monoclonal antibodies
**IFNs**	Interferons
**PDGF**	Platelet-derived growth factor
**Epo**	Erythropoietin
**RBP4**	Retinol-binding protein 4
**HIF-1α**	Hypoxia inducible factor-1 alpha
**VHL**	Von Hippel–Lindau
**siRNA**	Small interfering RNAs
**ODNs**	Decoy oligonucleotides
**HCC**	Hepatocellular carcinoma
**Shh**	Sonic hedgehog
**Ihh**	Indian hedgehog
**Dhh**	Desert hedgehog
**PTCH**	Patched
**SMO**	Smoothened
**SUFU**	Suppressor of fused
**Hhat**	Hedgehog acyltransferase
**RARα2**	Retinoic acid receptor α2
**SPOP**	Speckle-type POZ
**VASH2**	Vasohibin 2
**DOX**	Doxorubicin
**Arrb1**	β-arrestin1
**BCC**	Basal cell carcinoma
**GANTs**	Gli antagonists
**BPPs**	Balanophora polyandra Griff
**PIGF**	Placental growth factor
**VEGFRs**	Vascular endothelial growth factor receptors
**NRP1**	Neuropilin1
**ECM**	Extracellular matrix
**COUP-TFII**	COUP transcription factor II
**MLT**	Melatonin
**TRK**	Tyrosine kinase
**RCC**	Renal cell carcinoma
**R-Smads**	Receptor-regulated Smads
**FAK**	Focal adhesion kinase
**NK**	Natural killer
**CUG**	Cancer upregulated gene
**PTHrP**	Parathyroid thyroid hormone-related peptide
**ASOs**	Antisense oligonucleotides
**SMIs**	Small molecule inhibitors
**RT**	Radiotherapy
**TGFβR2**	TGFβ receptor 2
**subQ**	Subcutaneous
**R1-Ki**	TGF-β R1 kinase inhibitor
**PMNCs**	Peripheral blood mononuclear cells
